# SPHINX31 acts as a SRPK1 inhibitor targeting the ATR/DNA‐PKcs/CHK1 replicative checkpoint to inhibit cell growth in non‐small cell lung cancer

**DOI:** 10.1002/1878-0261.70339

**Published:** 2026-09-17

**Authors:** Amani Shreim, Aurelie Genoux, Nadiia Zubchuk, Haoyang Zheng, Perla Salameh, Theo Ziegelmeyer, Fabien Dalonneau, Margot Rouchette, Christiane Oddou, Simona Miron, Helene Polveche, Adiilah Mamode Cassim, Carmen Garrido, Eleni Nikolakaki, Didier Auboeuf, Tao Jia, Sophie Zinn‐Justin, Beatrice Eymin

**Affiliations:** ^1^ University Grenoble Alpes, INSERM U1209, CNRS UMR5309, Team RNA Splicing, Cell Signaling and Response to Therapies, Institute For Advanced Biosciences Grenoble France; ^2^ Institute for Integrative Biology of the Cell (I2BC) Université Paris‐Saclay, CEA, CNRS Gif‐sur‐Yvette France; ^3^ University Grenoble Alpes, INSERM U1209, CNRS UMR5309, Team INVADE, Institute For Advanced Biosciences Grenoble France; ^4^ I‐Stem, CECS Corbeil‐Essonnes France; ^5^ Laboratoire de Biologie et de Modélisation de la Cellule (LBMC), Ecole Normale Supérieure, CNRS UMR5239, INSERM U1293 Lyon France; ^6^ Université Bourgogne Europe, INSERM UMR123, Centre de Lutte Contre le Cancer Georges François Leclerc Dijon France; ^7^ Department of Chemistry Aristotle University of Thessaloniki, University Campus Thessaloniki Greece; ^8^ National Health Commission (NHC) Key Laboratory of Nuclear Technology Medical Transformation, Sichuan Provincial Engineering Research Center of Nuclear Medical Equipment Translation and Application Mianyang Central Hospital Mianyang China

**Keywords:** ATR, NSCLC, platinum salts, replicative stress, SPHINX31, SRPK1

## Abstract

Splicing targeting drugs have emerged as promising anticancer drugs. However, their mechanisms of action remain largely unknown, notably in Non‐Small Cell Lung Carcinoma (NSCLC). In this study, we demonstrated that SPHINX31, which targets SRPK1, inhibits the ATR/CHK1 signaling pathway, a cornerstone of the replicative stress response, leading to decreased cell proliferation, increased DNA damage and apoptosis, notably in NSCLC cells resistant to platinum salts. Mechanistically, we found that SRPK1 is recruited at stalled replication forks upon replicative stress, co‐immunoprecipitates with the ATR/ATRIP/TOPBP1 complex, directly interacts with TOPBP1 BRCT4/5 and BRCT7/8 domains, and contributes to the accumulation of TOPBP1 nuclear foci. We further showed that SRPK1 controls the splicing of genes previously related to ATR signaling, notably *WIZ*. All these events are prevented by SPHINX31. Lastly, we showed that the inhibitory effects of SPHINX31 on ATR are counterbalanced by the activation of DNA‐PKcs. Altogether, this study uncovers SRPK1 as a new component of the ATR/DNA‐PKcs/CHK1 replicative checkpoint. SRPK1 inhibitors, alone or in combination with DNA‐PKcs or CHK1 inhibitors, could provide therapeutic benefit in NSCLC patients including those who relapse after platinum‐based chemotherapy.

## Graphical abstract

The kinase SRPK1 directly interacts with the protein TOPBP1 and regulates the pre‐mRNA splicing of *WIZ* thereby contributing to the activation of the ATR/CHK1 replicative checkpoint in response to replicative stress. This allows cancer cells' genomic stability and survival. Pharmacological inhibition of SRPK1 might represent a new therapeutic strategy, notably in lung cancer cells with secondary resistance to platinum salts.

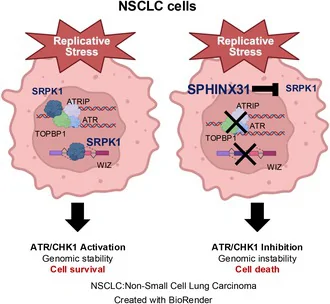

**Abbreviations**
53BP1: p53‐binding protein 1
AAD: ATR activation domain
ATM: ataxia telangiectasia mutated
ATR: ataxia telangiectasia and Rad3 related
ATRIP: ATR‐interacting protein
BCLAF1: BCL2‐associated transcription factor 1
BLM: bloom syndrome protein
BRCT: BRCA1 C terminus
CHK1: checkpoint kinase 1
CHK2: checkpoint kinase 2
DBF4B: DBF4B‐CDC7 kinase regulatory subunit
DNA‐PKcs: DNA‐dependent protein kinase catalytic subunit
DSB: DNA double strand break
ETAA1: Ewing's tumor‐associated antigen 1
FBS: fetal bovine serum
FGF‐2: fibroblast growth factor‐2
HU: hydroxyurea
LBR: lamin B receptor
NSCLC: non‐small cell lung cancer
PCNA: proliferating cell nuclear antigen
RFC5: replication factor C subunit 5
RPA: replication protein A
siRNA: small interfering RNA
SRPK1: SR protein kinase 1
ssDNA: single‐strand DNA
TERF1: telomeric repeat binding factor 1
THRAP3: thyroid hormone receptor‐associated protein 3
TOPBP1: DNA topoisomerase II‐binding protein 1
WIZ: widely interspaced zinc finger

## 1 Introduction

Lung cancer is the most common cause of cancer death worldwide [1]. Non‐Small Cell Lung Carcinoma (NSCLC) histology, including lung adenocarcinoma, represents approximately 85% of patients [2]. Platinum salts (cisplatin, carboplatin) are the gold standard chemotherapy in advanced NSCLC patients without targetable mutations [3]. However, the response rates remain below 30%, due to innate/acquired resistance and rate‐limiting side effects such as nephrotoxicity [4]. Therefore, we urgently need to identify new drugs to improve outcome in platinum salts‐resistant NSCLC patients. Pre‐mRNA splicing is a key post‐transcriptional step in the regulation of gene expression [5]. High‐throughput RNA‐sequencing analyses have now provided evidence that dysregulation of RNA splicing is a hallmark of cancer [6], with tumors, such as NSCLC, harboring up to 30% more alternative splicing events than paired normal tissue from the same individual [7]. These observations have provided the rationale for the development of small pharmacological compounds targeting components of the spliceosome machinery in cancer [8, 9, 10]. However, despite encouraging preclinical results for some of these molecules, we clearly need to improve basic knowledge regarding their mechanisms of action.

The SR Protein Kinase 1 (SRPK1) is an important regulator of the spliceosome machinery belonging to the serine–arginine protein kinase family that phosphorylates the Ser‐Rich (SR) splicing factors and regulates the pre‐mRNA splicing process [11, 12]. SRPK1 has been shown to contribute to various cancer hallmarks [13] and is overexpressed in a multitude of cancer types [11], including breast, colon, pancreatic carcinoma [14] or ovarian cancer [15]. We previously demonstrated that SRPK1 is upregulated in NSCLC [16]. We further showed that SRPK1 contributes to endothelial cells' proliferation and migration in response to Fibroblast Growth Factor‐2 (FGF‐2), thereby supporting angiogenesis and tumor progression in NSCLC [17]. According to the therapeutic potential of SRPK1 targeting in cancer, small molecule inhibitors which bind to SRPK1's ATP pocket have been developed [18], such as SRPIN340 or SCO‐101 [19, 20]. SPHINX31 is another ATP‐competitive inhibitor that shares the same scaffold with SRPIN340 but exhibits higher activity and selectivity for SRPK1 over SRPK2, and a lower IC_50_ value than that of SRPIN340 [21, 22]. SPHINX derivatives have been tested in preclinical studies in leukemia and solid tumors (colon, breast) [23, 24, 25], but their effects in NSCLC are currently not known.

Interestingly, SRPK1 has been controversially related to either cisplatin sensitivity or resistance in ovarian, testicular, pancreatic, colon, or breast carcinoma [14, 15, 26, 27, 28, 29, 30, 31]. These opposite effects could rely on the cellular context, the primary sensitivity of cells to cisplatin [15, 26], or SRPK1 subcellular distribution between the cytoplasm and the nucleus [12]. Hence, nuclear accumulation of SRPK1 correlated with 5‐fluorouracil and cisplatin sensitivity in HeLa and T24 cells [32]. In contrast, nuclear accumulation of SRPK1 in MCF7 and MDA‐MB‐231 breast cancer cells with secondary resistance to cisplatin was found to contribute to the resistant phenotype [31]. Up to now, the nuclear functions of SRPK1 have been primarily associated with the regulation of splicing events, through the control of SR proteins phosphorylation [33, 34]. However, SRPK1 can also phosphorylate other nuclear proteins, such as the Lamin B Receptor (LBR) which binds heterochromatin [35, 36]. In addition, SRPK1 has been shown to regulate transcription after UV radiation, possibly through interaction with the tumor‐associated proteins Bcl2‐Associated Transcription Factor 1 (BCLAF1) and Thyroid Hormone Receptor‐Associated Protein 3 (THRAP3) which bind RNA Polymerase II [37]. Therefore, nuclear SRPK1 appears to exhibit both splicing‐dependent and splicing‐independent functions, the last ones remaining to be further deepened.

In NSCLC cell lines, we previously found that the knock‐down of SRPK1 using siRNA increases cisplatin‐induced apoptosis, thereby indicating that SRPK1 could contribute to primary resistance to platinum salts in NSCLC [38]. In this study, we investigated the effects of pharmacological inhibition of SRPK1 using SPHINX31 in NSCLC cells, including cells with acquired resistance to platinum salts. Our results demonstrated that SPHINX31 inhibits Ataxia Telangiectasia and Rad3 related (ATR)/Checkpoint Kinase 1 (CHK1) signaling, the main pathway involved in the management of replicative stress, notably in resistant cells. Mechanistically, we demonstrated that SRPK1 is recruited at nascent replication forks upon replicative stress, is part of the ATR/ATR‐Interacting Protein (ATRIP)/DNA Topoisomerase II Binding Protein 1 (TOPBP1) complex, promotes ATRIP and TOPBP1 recruitment to chromatin, directly interacts with TOPBP1 4/5 and 7/8 BRCA1 C‐terminal (BRCT) domains, and contributes to TOPBP1 nuclear foci accumulation. This likely participates in ATR full activation. We also demonstrated that SRPK1 regulates the splicing of *WIZ* which has been previously involved in the replicative stress response [39, 40, 41]. As a whole, these results unravel a new nuclear function of SRPK1 in the management of replicative stress. We propose that cotargeting SRPK1 and components of the replicative stress checkpoint could provide therapeutic benefit in NSCLC patients, including those who relapse after platinum‐based chemotherapy.

## 2 Materials and methods

### 2.1 Cells, cell culture, reagents, and plasmids

Human NSCLC cell lines H460 (RRIDs: CVCL_0459); A549 (RRIDs: CVCL_0023) were authenticated by DNA STR profiling (ATCC cell line Authentication Service, LGC standards, Molsheim, France) and routinely tested for mycoplasma contamination. Parental and platinum salts‐resistant cells were derived from H460 NSCLC cells obtained from ATCC according to [42]. H460 resistant cells (annotated hereafter H460R) were less responsive to cisplatin‐induced cell growth inhibition and apoptosis compared to parental cells (annotated hereafter H460S) (Fig. S1A,B). A549 parental and platinum salts‐resistant NSCLC cells were obtained from Dr Martin Dutertre (Institut Curie, Paris). Resistance to cisplatin was confirmed by MTS (Fig. S1C) and clonogenic (Fig. S1D) assays. Parental and platinum salts‐resistant CAL33 Head and Neck Squamous Carcinoma (HNSCC) cells were obtained from Drs Maeva Dufies and Gilles Pagès (IRCAN, Nice) [43]. The resistance of these cells to cisplatin was characterized in Hagege et al. [43]. All platinum salts‐resistant models represent non clonal bulk of resistant cells. Cells were cultured in RPMI‐1640 medium/L‐glutamax supplemented with 10% (v/v) Fetal Bovine Serum (FBS) as previously described [44]. Platinum salts‐resistant H460 cells were continuously grown in presence of 20 μm carboplatin. In all experiments, carboplatin was removed before seeding. Carboplatin (cat#S1215; Selleckchem, Planegg, Germany) was dissolved in water at a stock solution of 10 mm. Cisplatin (cat#S1166; Selleckchem) was dissolved in dimethylformamide (DMF) at a stock solution of 50 mm. SPHINX31 at 10 mm in dimethylsulphoxide (DMSO) (cat#HY‐117661) was obtained from CliniSciences (Nanterre, France). SRPIN340 (cat#TA‐T1954) and VE‐821 (cat#TA‐T3032) at 10 mm in DMSO were obtained from Euromedex, NU7441 (KU‐57788) (cat#S2638), nedisertib (cat#1637542‐33‐6), CHK1 inhibitor (TCS2312, cat#838823‐31‐7) were obtained from Selleckchem, TargetMol and BioTechne®, respectively. Hydroxyurea (cat#H8627) was obtained from Sigma‐Aldrich (Saint Quentin Fallavier, France).

### 2.2 RNA interference

All the siRNAs were purchased from Eurogentec (Liege, Belgium). The two sequences designed to specifically target human *SRPK1* RNAs (Srpk1_1 and Srpk1_2) were as follows: 5′‐GCUAAUGACUGUGAUGUCCAAAA‐3′ and 5′‐CCAUGUGAUCCGAAAGUUAGG‐3′. In all RNA interference experiments, *control* siRNA was 5′‐UCGGCUCUUACGCAUUCAA‐3′. Cells were transfected with siRNA oligonucleotides duplex using RNAiMax reagent (Thermo Fisher Scientific, Waltham, USA) according to the manufacturer's instructions. The cells were analyzed 72‐h post‐transfection.

### 2.3 MTS and clonogenic survival assays, 3D culture

Cells (5 × 10^3^) were seeded in 96‐well plates and allowed to adhere overnight at 37 °C. Following 96‐h treatment of cells with increasing concentrations of SPHINX31 or cisplatin, MTS reagent [3‐(4,5‐dimethylthiazol‐2‐yl)‐5‐(3‐carboxymethoxyphenyl)‐2‐(4‐sulfophenyl)‐2H‐tetrazolium] was added to each well and incubated for 4 h at 37 °C. Absorbance was recorded at 490 nm. For clonogenic survival assay, 200 cells were seeded in 6‐well plates and treated or not with 5–10 μm SPHINX31 combined or not with 2 μm NU7441 or 1 μm CHK1 inhibitor for 14 days. In control DMSO condition, DMSO was added at 1 : 1000–1 : 2000 dilution in each well. Colonies were fixed in 4% glutaraldehyde for 5 min, washed two times in 1× Phosphate‐Buffered Saline (PBS), and stained with 1% methylene blue in 0.01 M sodium tetraborate decahydrate for 15 min at room temperature. Excess methylene blue was removed by washing with water and plates were dried overnight before colony counting. Spheroids were generated by seeding 1000 cells per well in 5 wells/condition in complete medium into 96‐well round bottom ultra‐low attachment (ULA) spheroid microplates (Corning, Tewksbury, MA, USA). After 4 days, spheroids were treated or not (Ctrl Medium) with DMSO (1 : 1000–1 : 2000; Ctrl DMSO), SPHINX31 and/or nedisertib and let grow. Same treatments were repeated at day 11. Spheroid formation and growth at days 4, 7, 11, and 14 were assessed on an inverted microscope using metamorph software and multidimensional acquisition. Spheroid area in μm^2^ was calculated for each condition using imagej software. The CellTiter‐Glo^R^ 3D cell viability assay (Promega, Charbonnière Les Bains, France) was used to determine the number of viable cells inside spheroids according to the manufacturer's instructions. Briefly, 100 μL of Ultra‐Glo™ recombinant luciferase was added in each well and the plate was incubated at room temperature for 25 min before luciferase quantification using Clariostar instrument.

### 2.4 Active caspase‐3 detection and quantification of bromodeoxyuridine (BrdU) incorporation by flow cytometry

Parental and resistant H460 cells were treated or not for 24 h with 10 μm SPHINX31. Medium and/or DMSO at 1 : 1000 dilution were used as controls. Detection of active caspase‐3 was performed by flow cytometry on an Accuri C6 flow cytometer (BD Biosciences) after cell fixation in Cytofix/Cytoperm™ and labelling with the phycoerythrin‐conjugated monoclonal active caspase‐3 antibody (BD Biosciences, Le Pont de Claix, France). Quantification of BromodeoxyUridine (BrdU) incorporation was performed using the APC BrdU Flow kit from BD Pharmingen™ (cat#sc‐552 598) according to the manufacturer's instructions. Briefly, treated or untreated parental and resistant H460 cells were incubated with BrdU for 1 h at 37 °C in full medium before stopping the experiment. Cells were washed, fixed and permeabilized in BD Cytofix/Cytoperm buffer, and stained for BrdU and 7‐AAD before flow cytometry analysis. Data from 10.000 cells were recovered and analyzed using the cellquest software (BD Biosciences).

### 2.5 Subcellular fractionation

All preparations were conducted on ice. For the preparation of cytoplasmic and nuclear fractions, cells were incubated for 15 min in hypotonic solution [10 mm Tris/HCl pH 7.5, 10 mm KCl, 1.5 mm MgCl_2_, 0.5 mm dithiothreitol (DTT)] supplemented with phosphatase and protease inhibitors just before use. The solution was centrifuged at 2000 rpm for 10 min, and the pellet was resuspended in hypotonic buffer containing 0.9% Nonidet P40 (NP40), then incubated for 10 min. The supernatant (cytoplasmic fraction) was collected after centrifugation at 1200 rpm for 15 min, the pellet was washed two times in 1× PBS and centrifuged at 600 rpm for 5 min to obtain a white and viscous pellet. Then, the pellet was resuspended in radio‐immunoprecipitation (RIPA) buffer [50 mm Tris/HCl pH 7.5, 150 mm NaCl, 0.1% sodium dodecyl sulfate (SDS), 1% NP40, 0.5% sodium deoxycholate] supplemented with phosphatase and protease inhibitors and incubated for 30 min (vortexed every 5 min), sonicated for 5 min, centrifuged at 13,200 rpm for 15 min at 4 °C, and the supernatant (nuclear fraction) was collected and stored at −80 °C.

### 2.6 Cellular protein extracts and chromatin‐enriched fractions

For cellular protein extraction, cells washed three times in 1× PBS were lysed in RIPA buffer for 30 min on ice and pelleted by centrifugation at 13,200 rpm for 20 min at 4 °C. Chromatin‐enriched fractions were prepared according to [45]. Briefly, 2 × 10^6^ cells were incubated 10 min on ice in a hypotonic buffer containing 10 mm 4‐(2‐hydroxyethyl)piperazine‐1‐ethanesulfonic acid (HEPES), pH 7.9, 1.5 mm MgCl_2_, 46.7 mm sucrose, 10% glycerol, 0.1% Triton, and 1 mm DTT supplemented just before use with phosphatase and protease inhibitors and centrifuged 4 min at 1300 rpm. Supernatants (fraction S1) were eliminated and cell nuclei were incubated 10 min on ice in a buffer containing 3 mm ethylenediaminetetraacetic acid (EDTA), 0.2 mm egtazic acid (EGTA), 1 mm DTT with phosphatase and protease inhibitors before centrifugation at 1700 rpm for 4 min. Supernatant (fraction S2 containing soluble nuclear proteins) was eliminated and the cell pellet containing nuclear proteins tightly bound to chromatin (P2 extracts, chromatin‐enriched fractions) was resuspended in NuPAGE Buffer (Invitrogen™, cat#NP0007) and sonicated.

### 2.7 Immunoblotting and immunoprecipitation

Immunoblotting experiments were performed as previously described [46]. Antibodies used in this study are listed in Table S1. For immunoprecipitation, H460 resistant cells were treated or not with 10 μm SPHINX31 and/or 2 mm hydroxyurea (HU) for 24 h. Cells were counted and 2 × 10^6^ cells per condition were used to perform subcellular fractionation as described above. Chromatin‐enriched fractions were resuspended in TNE buffer (50 mm Tris/HCl pH 7.4, 150 mm NaCl, 1% Triton X‐100, 1 mm EDTA, 1 mm EGTA, 0.5% NP40) supplemented with protease and phosphatase inhibitors and 1 μL benzonase. Anti‐TOPBP1 (Cell Signaling Technology, cat#14342) was incubated for 10 min in presence of protein G‐magnetic beads (Dynabeads™, cat#10003D), then incubated with chromatin‐enriched fractions overnight at 4 °C. Beads‐antibody complexes were washed 3 times in NET buffer (50 mm Tris/HCl pH 7.5, 150 mm NaCl, 1 mm EDTA, 0.1% NP40, 0.02% sodium azide), and resuspended in NuPAGE buffer (Thermo Fisher Scientifics) before loading. An irrelevant rat IgG1 isotype was used as control for immunoprecipitation.

### 2.8 Western blot in capillary using JESS

Levels of total ATR and phospho‐ATR(Thr1989) proteins were analyzed using the capillary‐based instrument JESS Simple Western™ (ProteinSimple®, BioTechne). Based on the expected molecular weights of the proteins, a 66–440 kDa separation module with 25 capillary cartridges (SM‐W008) was used. Following optimization of antibody dilution (ATR antibody: Cell Signaling Technology, cat#2790, dilution 1 : 10; phospho‐ATR(Thr1989) antibody: Cell Signaling Technology, cat#30632, dilution 1 : 10) and sample concentration (0.5 μg·μL^−1^), the assays were conducted according to the manufacturer's protocol. Samples were diluted in 0.1× sample buffer (cat#042‐195), mixed with the Fluorescent Master Mix (cat#PS‐ST03EZ‐8) in a 4:1 ratio, heated at 95 °C for 5 min, and then 5 μL of each sample was added per well. The ready‐to‐use anti‐rabbit secondary horseradish peroxidase (HRP)‐conjugated antibody (DM‐001) and luminol‐peroxide reagents were used following the kit's instructions (anti‐rabbit detection module chemiluminescence, ProteinSimple®). Replex™ (RP‐001) and Total Protein Detection (DM‐TP01) modules were used to normalize each plate. Data were analyzed using Compass for simple western software v5.0.0. For normalization, the resulting area of each specific peak was divided by the total protein value of the respective antibody.

### 2.9 ‘*In vitro*’ SRPK1 kinase assay

SRPK1 kinase assay was performed as described previously [32]. Briefly, the assays were carried out in a total volume of 25 μL containing Glutathione‐S Transferase (GST)‐TOPBP1(978–1192), GST‐TOPBP1(978–1522) or GST‐Lamin B Receptor (LBR)‐Nt(62–92) recombinant protein (2 μg each) as the substrate and GST‐SRPK1 (0.5 μg) with 25 μm adenosine triphosphate (ATP), 1 μCi of [γ‐^32^P]ATP for 30 min at 30 °C. Phosphoproteins were detected with autoradiography using Super RX (Fuji medical X‐ray film, Fujifilm, Düsseldorf, Germany).

### 2.10 NMR‐based phosphorylation assay

For nuclear magnetic resonance (NMR) analysis, a ^15^N‐labeled TOPBP1 fragment (residues 1055–1177) was produced in BL21 (DE3) Star grown in M9 medium containing 0.5 g·L^−1 15^NH_4_Cl and 2 g·L^−1 12^C‐glucose and purified by affinity chromatography. 2D NMR ^1^H‐^15^N SO‐FAST HMQC spectra were recorded using a Bruker 700 MHz spectrometer on the ^15^N labeled TOPBP1 fragment (50 μm, in 50 mm Hepes pH 7.0, 50 mm NaCl, 1 mm DTT) either alone or after incubation during one night at 25 °C with the full‐length SRPK1 recombinant protein (cat#PV4215; Thermo Fisher Scientific, CA, USA) (0.14 μm), 20 mm MgCl_2_ and 5 mm ATP.

### 2.11 Expression and purification of TOPBP1 BRCT4/5 and 7/8

TOPBP1 BRCT4/5 (559–746) and TOPBP1 BRCT7/8 (1264–1493) fragments were expressed from pGEX‐6P‐1 plasmids kindly provided by J. N. Mark Glover. The plasmids were transformed into *E. coli* BL21 (DE3) Star cells (Thermo Scientific, EC0114), which were grown at 37 °C to an OD_600_ of 0.8. Protein expression was induced with 0.2 mm isopropyl‐β‐d‐thiogalactopyranoside (IPTG), followed by overnight incubation at 20 °C. Cells were harvested by centrifugation and resuspended in 25 mm Tris/HCl (pH 7.5), 150 mm NaCl, 5 mm β‐mercaptoethanol, 10% glycerol, and then lysed by sonication (Vibra‐Cell™, VC 505) with proteases inhibitors (complete™, EDTA‐free Protease Inhibitor Cocktail, Roche). Proteins were purified as previously described [47, 48].

### 2.12 Biolayer interferometry (BLI)

The interactions between a biotinylated SRPK1 N‐terminal peptide (either biotinylated and nonphosphorylated or phosphorylated at Ser51) and TOPBP1 BRCT4/5 (549–746) and BRCT7/8 (1264–1493) were analyzed at 25 °C using an Octet RED96 biolayer interferometry (BLI) instrument (ForteBio). The peptides (Biotin‐GSG‐^43^EQEEEILGSDDDEQ^56^ and Biotin‐GSG‐^43^EQEEEILGpSDDDEQ^56^, where pS denotes a phosphorylated serine) were synthesized by Genecust and immobilized on streptavidin (SA) biosensors prehydrated for 20 min in 25 mm Hepes (pH 7.0), 50 mm NaCl, 5 mm β‐mercaptoethanol buffer. No nonspecific binding was detected between the SA biosensors and either TOPBP1 BRCT4/5 or TOPBP1 BRCT7/8 proteins. A solution of 0.5 μm biotinylated peptides was used for immobilization on SA biosensors. Biosensors were exposed to increasing concentrations of TOPBP1 BRCT 7/8 (0.2, 0.4, 0.8, 1.6, and 3.2 μm) or TOPBP1 BRCT 4/5 (0.2, 0.4, 0.8, 1.6, and 3.2 μm for the phosphorylated peptide and 1.48, 2.95, 5.9, 11.8, and 23.6 μm for the nonphosphorylated peptide). Following association made with the TOPBP1 fragments dissolved in the 25 mm HEPES, pH 7, 50 mm NaCl, 5 mm β‐mercaptoethanol, and 0.05% Tween‐20 buffer, biosensors were transferred to the same buffer for dissociation measurements. Each measurement cycle consisted of a baseline (120 s), association (300 s), and dissociation (300 s) phase. Regeneration between cycles was performed using 1 M NaCl. Equilibrium dissociation constants (*K*d) were determined using the steady‐state analysis.

### 2.13 Immunofluorescence studies

Immunofluorescence analyses of S‐phase replicating cells were performed using click‐iT EthynyldeoxyUridine (EdU) imaging kit (cat#C10340; Thermo Fisher Scientific) according to the manufacturer's instructions. Briefly, 50 × 10^3^ parental and platinum salts‐resistant H460 cells were seeded onto 18‐mm round coverslips precoated with 5 μg·mL^−1^ fibronectin (cat#F2006; Sigma‐Aldrich), treated or not for 24 h with 10 μm SPHINX31, and incubated with 10 μm EdU for 1 h at 37 °C. Cells were then fixed with 4% paraformaldehyde (PFA) for 15 min at RT, permeabilized in 0.5% Triton for 20 min at RT, before performing the Click‐iT reaction using Alexa Fluor™ 647‐azide. Cells were then stained with anti‐γH2AX antibody (cat#2577; Cell Signaling), and nuclei were counterstained using 6‐diamidino‐2‐phenylindole (DAPI). For quantification of γH2AX and 53BP1 nuclear foci, H460R cells were grown on glass coverslips in a 12‐well plate, rinsed three times with 1× PBS, and fixed on the coverslips with 10% formalin for 12 min. Next, the cells were rinsed three times with 1× PBS, permeabilized with 0.5% Triton for 5 min, and then rinsed again three times with 1× PBS. Subsequently, 3% PBS‐BSA (bovine serum albumin) was added to the cells for 30 min, after which the solution was removed, and a solution of 3% 1× PBS‐BSA with γH2AX (clone JBW301; Sigma‐Aldrich) or 53BP1 antibody was added. After incubation for 2 h or overnight, the cover slips were washed four times with 3% 1× PBS‐BSA, and a solution of 3% 1× PBS‐BSA with secondary antibody was added. Next, coverslips were placed in the dark. After 2 h, cells were rinsed three times with 1× PBS, a drop of DAPI‐Vectashield (Roche) was placed on a microscope slide, and the coverslip was placed on top of it. Cells were observed using an Olympus microscope (×63 magnification). Images were captured with a Coolview CCD camera (Photonic Science) and digitally saved using visilog software. The number of γH2A.X foci was analyzed using imagej software and the macro Analyze_foci_in_nuclei_fixed_4_61.ijm developed by Bram van den Broek (NKI institute, The Netherlands).

For immunofluorescence of TOPBP1 foci, 50 × 10^3^ cells were seeded onto 18 mm round coverslips precoated with 5 μg·mL^−1^ fibronectin, treated or not for 24 h with 10 μm SPHINX31 in the presence or absence of 2 mm hydroxyurea, incubated for 3 min at 4 °C in cytoskeleton (CSK) buffer [10 mm 1,4‐piperazinediethanesulfonic acid dipotassium salt (PIPES) pH 7, 100 mm NaCl, 300 mm sucrose, 3 mm MgCl_2_, 0.2% Triton X‐100, protease and phosphatase inhibitors], fixed in 4% PFA for 15 min at RT, permeabilized in 0.2% Triton for 10 min at RT, blocked in 3% bovine serum albumin (BSA), 0.1% Tween in 1× PBS for 1 h at room temperature (RT), incubated with 1 : 200 TOPBP1 antibody (cat#sc‐271 043; Santa Cruz), washed in 0.1% Tween in 1× PBS and incubated with secondary goat anti‐mouse antibody Alexa Fluor™ 647 (cat#A‐21241; Thermo Fisher Scientifics) 1 : 500 in 3% BSA/1× PBS for 1 h at RT in dark before slides mounting in Prolong™ Gold Antifade mountant with DAPI. Quantification of corrected TOPBP1 fluorescence/nucleus (CTCF) was performed using imagej software. Regions of interest (ROI) were defined as nucleus of each cell and the CTCF was calculated using the formula: CTCF = Integrated Density – (Area of selected cell × Mean Fluorescence of background reading).

For SRPK1 immunofluorescence studies, H460S and H460R cells were seeded at 5000 cells·cm^−2^ on glass coverslips and treated with 10 μm SPHINX31 for 6 to 24 h. Control conditions corresponded to treatment with DMSO for 24 h (1 : 1000). Cells were fixed in 10% formalin for 15 min, permeabilized with 0.2% Triton X‐100 in PBS for 15 min, and blocked 1 h in 1× PBS with 10% normal goat serum (NGS). SRPK1 antibody was diluted in PBST (0.05% Tween‐20) with 10% NGS and incubated for 1 h, followed by an incubation of 1 h in the dark with Alexa Fluor 488‐conjugated secondary antibody in the same buffer. Nuclei were stained with DAPI 1 μm for 15 min. Samples were washed, mounted (FluorSave, Millipore, ref. 345 789), and dried overnight at 4 °C. Images were acquired on an Apotome system with a 63× oil‐immersion objective. SRPK1 fluorescence intensity was quantified using CellProfiler (v3.1.1).

### 2.14 SIRF analysis

Quantitative ‘*in situ’* analysis of protein interactions at DNA replication forks (SIRF) was performed in parental and resistant H460 cells according to Roy et al. [49]. SIRF allows for the quantitative assessment of protein interactions with ongoing, stalled, and previously active replication forks using Proximity Ligation Assay (PLA) technology. Briefly, 2 × 10^4^ parental or 4 × 10^4^ resistant H460 cells grown in log‐phase were plated onto microscope chamber slides and treated or not with 5 μm SPHINX31 for 18 h or 2 mm hydroxyurea for 4 h. At the time of experiment, cells were incubated with 125 μm 5′‐ethylene‐2′‐deoxyuridine (EdU) for 10 min, washed two times in ice‐cold 1× PBS, permeabilized during 5 min at 4 °C in CSK buffer (10 mm PIPES pH 6.8, 100 mm NaCl, 300 mm sucrose, 3 mm MgCl_2_, protease inhibitors cocktail) containing 0.5% Triton X‐100 before fixation in 2% PFA for 15 min at room temperature. After fixation, PFA was discarded and the slides were extensively washed with 1× PBS before being incubated with the freshly prepared click reaction cocktail (2 mm copper sulfate, 9.5 μm biotin azide, 100 mm sodium ascorbate, 1 μm Alexa Fluor 488‐azide in 1× PBS) for one hour at room temperature. EdU (cat#A10044), Alexa Fluor 488‐azide (cat#A10266), and biotin azide (cat#B10184) were purchased from Thermo Fisher Scientific. After the click reaction, slides were blocked with blocking buffer containing 10% goat serum and 0.1% Triton X‐100 in 1× PBS for 1 h at RT and incubated at 4 °C overnight with the primary antibodies. The primary antibodies used in SIRF were: rabbit anti‐biotin (cat#A150‐109A; Bethyl Laboratory; 1/200e), mouse anti‐PCNA (cat#sc‐56; Santa Cruz Technology; 1/200e), or mouse anti‐SRPK1 (cat#611072; BD Biosciences; 1/200e). PLA was performed by incubating the slides for 1 h at 37 °C in the presence of Duolink Rabbit minus and Duolink Mouse plus PLA probes (Sigma‐Aldrich) diluted 1 : 5 in blocking solution. After washes, slides were incubated in ligation mix containing Duolink ligation stock (1 : 5) and ligase (1 : 40) at 37 °C for 30 min. After washes, amplification was performed by incubating slides at 37 °C for 100 min in an amplification mix containing Duolink amplification stock (1 : 5) and rolling circle polymerase (1 : 80). After extensive washes, slides were mounted in Prolong™ Gold Antifade mountant with DAPI and analyzed using a multiphoton LSM510 META NLO confocal microscope (Zeiss, Oberkochen, Germany). Images were acquired with AxioCam digital microscope camera and analyzed using volocity software. All images are *z*‐stacked. SIRF controls included PLA with no click reaction or PLA‐EdU in cells cultured with 10 μm thymidine for 2 h after EdU incorporation (control chase).

### 2.15 Proximity ligation assay (PLA)

4 × 10^4^ H460R cells per well were seeded in Labteks. After 48 h, 10 μm SPHINX31 and/or 2 mm hydroxyurea were added for 24 h. Antibodies used were anti‐TOPBP1 (1 : 200) and anti‐SRPK1 (1 : 200). PLA was conducted as described above.

### 2.16 RNA extraction and RT‐PCR analysis

Total RNA was extracted using NucleoSpin RNA kit (Macherey Nagel) according to the manufacturer's instructions. About 1 μg total RNA was reverse transcribed using iScript™ Reverse Transcription Supermix (Bio‐Rad). The primers used were as follows: *GAPDH*‐Fw 5′‐CGA‐GAT‐CCC‐TCC‐AAA‐ATC‐AA‐3′; *GAPDH*‐Rv 5′‐ATC‐CAC‐AGT‐CTT‐CTG‐GGT‐GG‐3′; *DBF4B*‐Ex6‐Fw 5′‐TCT‐AAG‐CAG‐AGG‐GAA‐GGA‐GC‐3′; *DBF4B*‐Ex8‐Rv 5′‐CTG‐GCT‐TCT‐TTG‐GCT‐GTT‐GT‐3′; *RFC5*‐Ex1‐Fw 5′‐GCG‐ACC‐AAG‐ATC‐AGG‐AAC‐CT‐3′; *RFC5*‐Ex3‐Rv 5′‐GGG‐TCT‐GTG‐GCC‐GGT‐ATT‐TT‐3′; *WIZ*‐Ex7‐Fw 5′‐CAC‐AGT‐GGC‐CTC‐AGT‐CTG‐A‐3′; *WIZ*‐Ex9‐Rv 5′‐GAA‐CTC‐ACC‐ACA‐GAA‐CTC‐GC‐3′. PCR conditions were 94 °C, 2 min, 5 cycles (94 °C, 30 s; 64 °C, 30s; 72 °C, 1 min), 5 cycles (94 °C, 30 s; 61 °C, 30s; 72 °C, 1 min), 5 cycles (94 °C, 30 s; 59 °C, 30s; 72 °C, 1 min), and 25 cycles (94 °C, 30 s; 56 °C, 30s; 72 °C, 1 min). Amplicons were detected on 2% agarose gels in 1× Tris‐Borate‐EDTA (TBE).

### 2.17 RNA‐sequencing

Three biological replicate RNA samples from parental and resistant H460 cells treated or not with DMSO as a control or 5 μm SPHINX31 for 24 h were prepared using the NucleoSpin RNA isolation kit from Macherey‐Nagel. RNA‐Seq analysis was done by Novogen (Cambridge, United Kingdom). After RNA sample QC and directional library preparation (rRNA removal using Ribo‐Zero kit), reads were generated on an Illumina NovaSeq 6000 platform (150 bp Paired End) allowing to obtain 15 G raw data per sample. Reads were mapped and filtered using TopHat2 (2.0.13), Samtools (0.1.19) and PrinSeq (0.20.4). Splicing analysis was performed using FARLINE, a freely available computational program (http://kissplice.prabi.fr/pipeline_ks_farline) or rMATS that quantify alternative splicing variations [50]. The percent spliced in (PSI) value corresponding to exon inclusion rate was used to determine the sets of exons that are differentially spliced when comparing treated parental or resistant cells to untreated parental or resistant cells. Exons are defined as differentially regulated if the PSI variation (deltaPSI) is greater than 10% or lower than −10%, with *P*‐values ≤ 0.05, as compared to the corresponding control. Exons were defined using FASTERDB (http://fasterdb.ens‐lyon.fr/faster/home.pl). Gene Ontology Enrichment analysis was performed using the Gene Annotation and Analysis Resources Metascape [51].

### 2.18 Correlation studies using TCGA and CCLE databases

Expression values for *SRPK1, PRKDC, TOPBP1, ATR, RFC5*, and *DBF4B* mRNAs were retrieved from human tumors collected by Genomic Data Commons (GDC) TCGA Lung Adenocarcinoma (GDC TCGA LUAD). They were downloaded as log_2_ (FPKM‐UQ + 1) values using the UCSC Xena platform [52]. The correlation analysis was done by calculating Spearman's correlation coefficients and statistical significance using graphpad prism 8 software.

Protein expression levels of ATR (UniProt Accession: Q13535) and SRPK1 (UniProt Accession: Q96SB4) in lung cancer cell lines were retrieved from the Cancer Cell Line Encyclopedia (CCLE) proteomics dataset (https://gygi.hms.harvard.edu/publications/ccle.html). The data were downloaded as normalized protein quantification values. Spearman's correlation coefficients were calculated to assess the relationship between protein expression levels of the indicated targets across lung cancer cell lines. Statistical significance was evaluated by graphpad prism 8.

### 2.19 Statistical analysis

Analyses were performed using the graphpad prims software (GraphPad Software Inc., San Diego, CA, USA), and data are presented as mean ± SD. Statistical comparisons between two groups were conducted with Mann–Whitney, paired, or unpaired *t*‐test.

## 3 Results

### 3.1 SPHINX31 inhibits cell growth of NSCLC cells, including cells with acquired resistance to platinum salts

**Fig. 1:**
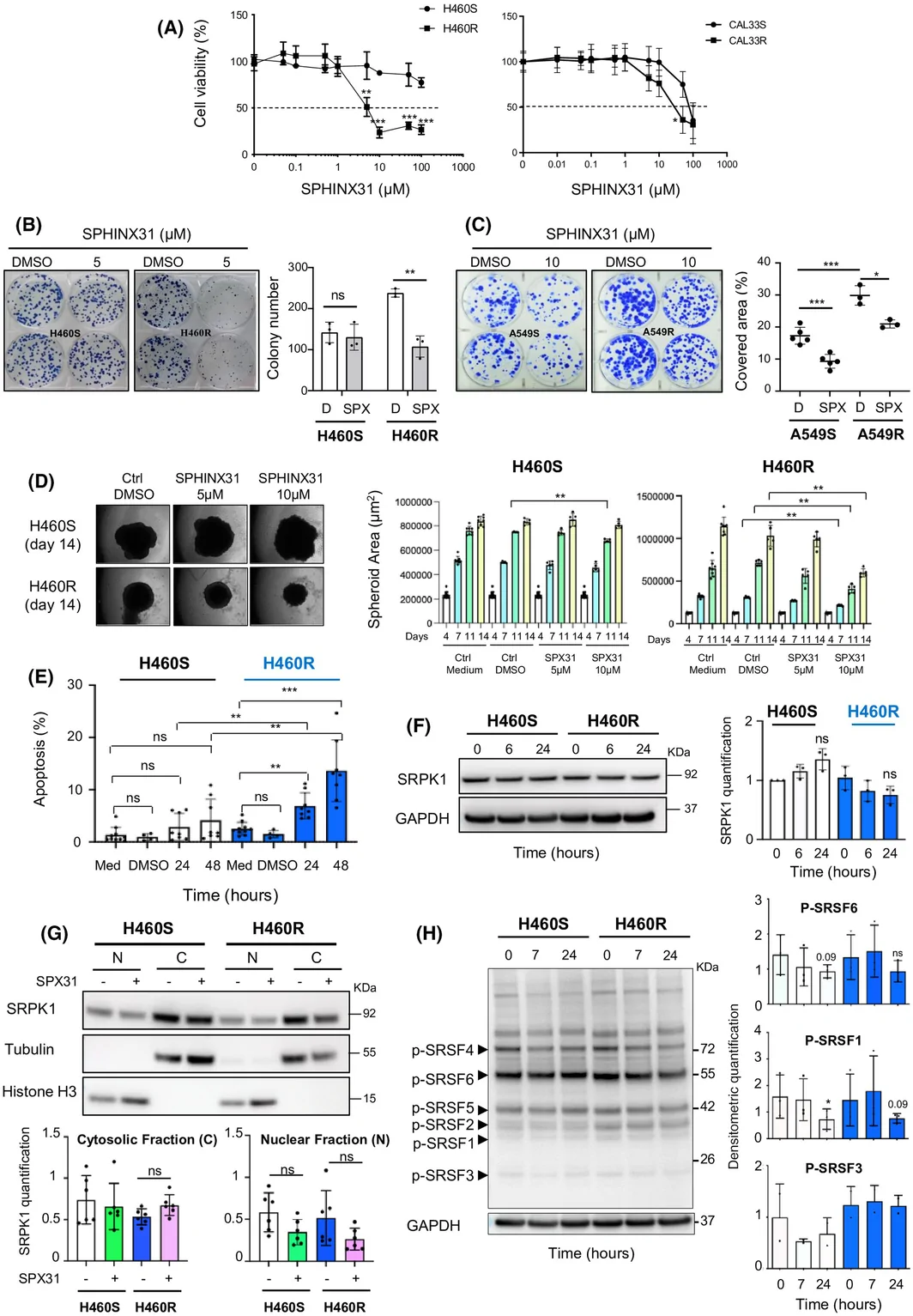
NSCLC cells with acquired resistance to platinum salts are vulnerable to SPHINX31. (A) MTS assay in H460S and H460R or Cal33S and Cal33R cells treated or not (medium) for 96 h with increasing concentrations of SPHINX31. Mean ± SD *n* = 3. (B) Clonogenic assay in H460S/R cells treated or not (DMSO) for 14 days with 5 μm SPHINX31. Right panel: quantification of the number of colonies. Mean ± SD *n* = 3. D: control DMSO. (C) Clonogenic assay in A549S/R cells treated or not (DMSO) for 14 days with 10 μM SPHINX31. Right panel: percentage of the covered area. Mean ± SD *n* = 3–4. D: control DMSO. (D) Spheroids from H460S/R cells were treated with complete medium or DMSO as control, or 5 μm or 10 μm SPHINX31 for the indicated days. Left panel: representative images at day 14. Middle and right panels: spheroids area in μm^2^. Mean ± SD. A representative experiment of 3 independent ones. *n* = 5 spheroids/condition. (E) Percentage of apoptosis in H460S/R cells treated or not (Med: medium only, DMSO) with 10 μm SPHINX31 for the indicated times. Mean ± SD *n* = 4–8. (F) Left panel: representative immunoblots of SRPK1 in H460S/R cells treated or not (medium) with 10 μM SPHINX31 for indicated times. GAPDH was used as a loading control. Right panel: densitometric quantification of SRPK1 signal normalized to GAPDH signal. Mean ± SD *n* = 3. (G) Upper panels: representative immunoblots of SRPK1 in cytosolic (C) and nuclear (N) fractions of H460S or R cells treated (+) or not (medium, −) with 10 μm SPHINX31 for 24 h. Tubulin and histone H3 were used as loading controls for cytosolic and nuclear fractions, respectively. Lower panels: densitometric quantification of SRPK1 signal normalized to tubulin or histone H3 signal. Mean ± SD *n* = 3. (H) Left panel: representative immunoblot of phospho‐SR proteins using 1H4 antibody in H460S/R cells treated or not (medium) with 10 μm SPHINX31 for indicated times. GAPDH was used as a loading control. Right panels: densitometric quantification of phospho‐SR proteins signal normalized to GAPDH signal. Mean ± SD *n* = 3. All unpaired *t*‐test. *P < 0.05, **P < 0.01, ***P < 0.001, ns: not significant.

We previously showed that siRNA‐mediated SRPK1 knock‐down enhanced cisplatin‐induced apoptosis in NSCLC cells [38]. In this study, we tested the effects of pharmacological inhibition of SRPK1 using SPHINX31, a highly selective SRPK1 inhibitor [23], in NSCLC cells, including cells with secondary resistance to cisplatin. In a 72 h MTS viability assay, H460‐derived resistant NSCLC cells (Fig. S1A,B) were found to exhibit higher sensitivity to SPHINX31 as compared to parental cells (Fig. 1A, left panel). CAL33 HNSCC cells with acquired resistance to platinum salts were also slightly more sensitive to SPHINX31 compared to parental cells (Fig. 1A, right panel). In a 14 days clonogenic survival assay, 5 μm SPHINX31 significantly reduced the number of colonies derived from H460R cells, while this was not observed in H460S cells (Fig. 1B). SPHINX31 also decreased the size of colonies derived from A549 NSCLC parental and platinum salts‐resistant cells, although at a higher concentration (Fig. 1C). SPHINX31 slowed down the growth of H460R‐derived spheroids, while it was less efficient on H460S‐derived spheroids (Fig. 1D). Moreover, as quantified by flow cytometry after active caspase‐3 staining, SPHINX31 enhanced apoptosis in H460R cells compared to H460S cells (Fig. 1E). As a whole, these results indicated that cancer cells with acquired resistance to platinum salts could be vulnerable to SPHINX31‐induced cell growth inhibition.

Then, in order to deepen the molecular mechanisms involved, we focused on H460S/R cells as a model. We previously demonstrated that SRPK1 accumulates in NSCLC tissues compared to normal lung [16]. We did not observe any variation of SRPK1 protein level between parental and resistant H460 cells, either at the basal level or upon SPHINX31 treatment (Fig. 1F). As nuclear translocation of SRPK1 has been associated with cisplatin sensitivity in HeLa and T24 cells [32], we performed subcellular fractionation followed by immunoblotting. We did not find differences as regard to SRPK1 nuclear/cytoplasmic distribution between parental and resistant cells (Fig. 1G). Although not significant, SPHINX31 tended to decrease the amount of nuclear SRPK1 in both cell lines (Fig. 1G). Immunofluorescence studies confirmed a decrease of SRPK1 nuclear staining upon SPHINX31 treatment in resistant cells while this was not seen in parental ones at the same time of treatment (Fig. S1E). As detected using the phospho‐SR antibody 1H4, SPHINX31 also tended to decrease the phosphorylation of some SR proteins in both cell lines (e.g., SRSF1, SRSF6), without any significant difference between H460R and H460S cells (Fig. 1H). These results suggested that the enhanced cytotoxic effects of SPHINX31 on H460R cells compared to parental ones do not strictly rely on increased inhibition of SR proteins phosphorylation.

### 3.2 SPHINX31 slows‐down cell cycle progression and leads to γH2AX accumulation in NSCLC cells with acquired resistance to platinum salts

**Fig. 2:**
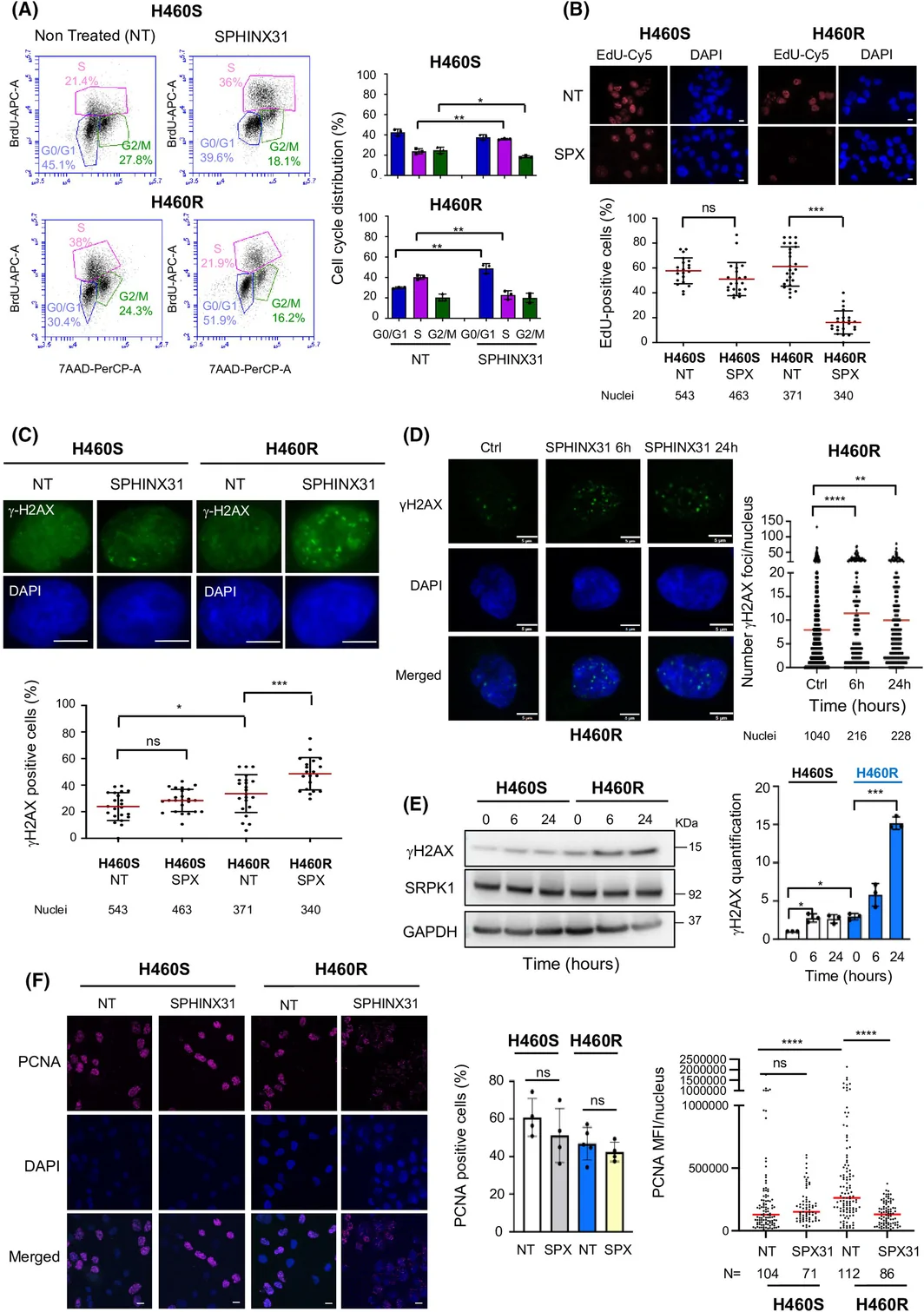
SPHINX31 slows‐down DNA replication which correlates with enhanced DNA damage and decreased recruitment of PCNA at nascent replication forks. (A–E) H460S or H460R cells were treated or not (medium, NT) with 10 μm SPHINX31 for 24 h (A–C) or the indicated times (D, E). (A) Left panels: representative experiment of Fluorescence‐Activated Cell Sorting (FACS) analysis of BrdU‐ and 7‐AAD‐A‐positive cells. Right panels: quantification of the cell cycle distribution (%). Mean ± SD, *n* = 3. (B, C) Upper panels: representative immunofluorescence staining of EdU‐Cy5 (B) or γH2AX (C). DAPI was used to counterstain the nucleus. Scale bar = 10 μm. Lower panels: quantification of the percentage of EdU‐positive cells (B) or γH2AX‐positive cells (with > 10 foci/nucleus, C). Each point represents the % of positive cells in 20–25 nuclei/field. (B, C) Mean ± SD *n* = 3 (> 100 nuclei counted/experiment). (D) Left panels: representative immunofluorescence staining of γH2AX nuclear foci at the indicated times. DAPI was used to counterstain the nucleus. Scale bar = 5 μm. Right panel: quantification of the number of γH2AX nuclear foci/nucleus. Mean. *n* = 3. Ctrl = DMSO. (E) Left panel: representative immunoblots of γH2AX and SRPK1. GAPDH was used as a loading control. Right panel: densitometric quantification of γH2AX signal normalized to GAPDH signal. Mean ± SD *n* = 3. (F) PCNA‐SIRF in H460S or H460R cells treated or not (medium, NT) for 18 h with 5 μm SPHINX31. Left panels: representative images of PCNA‐SIRF signals. Scale bar = 10 μm. Middle panel: percentage of PCNA‐SIRF‐positive cells. Mean ± SD *n* = 4–5. Right panel: corrected Mean Fluorescence Intensity (MFI) (mean ± SD) of PCNA‐SIRF signal/nucleus. 25–30 nuclei/experiment. In all experiments, unpaired *t*‐test. ****P < 0.0001, ***P < 0.001, **P < 0.01, *P < 0.05, ns: not significant.

To go further, we analyzed the effects of SPHINX31 on cell cycle distribution in H460S/R cells by two‐dimensional flow cytometry after costaining with 5‐bromo‐2′‐deoxyuridine (BrdU) and 7‐aminoactinomycin D (7‐AAD‐A). In H460R cells, a 24‐h treatment decreased the percentage of cells incorporating BrdU in S‐phase and increased the percentage of cells in G_1_ phase, while in H460S cells, it increased the percentage of cells incorporating BrdU in S‐phase and decreased the percentage of cells in G_2_/M (Fig. 2A). Immunofluorescence studies using Click‐It‐EdU assay confirmed a decrease in the percentage of EdU‐positive H460R cells after 24‐h treatment, while no significative effect was seen in H460S cells (Fig. 2B). Such a decrease of EdU incorporation in H460R cells was not seen at earlier time point (e.g., 7 h, Fig. S1F). In parallel, we analyzed γH2AX and p53‐Binding Protein 1 (53BP1), two well‐recognized markers associated with DNA double‐strand breaks (DSBs) [53, 54]. Immunofluorescence studies demonstrated increased percentage of H460R cells positive for γH2AX upon 24‐h treatment with SPHINX31, while this was not observed in H460S cells (Fig. 2C). SPHINX31 also increased the number of γH2AX nuclear foci/nucleus (Fig. 2D) and the percentage of cells exhibiting 53BP1 nuclear foci in H460R cells (Fig. S1G). These DSB markers were already detected after 6‐h treatment. Similar results were obtained when γH2AX protein level was analyzed by western blot (Fig. 2E). Noteworthy, untreated H460R cells accumulated more γH2AX compared to H460S cells as detected by immunofluorescence (Fig. 2C) or western blotting (Fig. 2E), thereby suggesting higher intrinsic level of DNA damage in these cells. Taken together, these results demonstrated that SPHINX31 slows‐down cell proliferation that correlated with decreased BrdU/EdU incorporation and enhanced DNA damage in H460 resistant cells.

These results led us to investigate whether SPHINX31 could promote replicative stress in H460R cells. To test this hypothesis, we performed *‘in situ’* analysis of protein interactions at DNA replication forks (SIRF) that allows us to quantify the recruitment of proteins at nascent replication forks [49]. We analyzed the recruitment of the DNA polymerase processivity factor, namely Proliferating Cell Nuclear Antigen (PCNA), a marker of active replication forks. For these SIRF experiments, we used a lower dose of SPHINX31 (5 μm) and a shorter time of exposure (18 h). First, we verified that SPHINX31 does not significantly impact the protein level of PCNA in parental or resistant cells (Fig. S2A). We also proved that the PCNA‐SIRF signal was well‐specific by doing several controls including SIRF without antibodies or with only one antibody (Fig. S2B), SIRF with no click reaction (Fig. S2C), or by performing pulse‐chase with thymidine before SIRF, which strongly reduced the PCNA‐SIRF signal (Fig. S2D). In these SIRF experiments, SPHINX31 did not significantly impact the percentage of PCNA‐positive cells in both H460S and H460R models (Fig. 2F, left and middle panels). In contrast, it significantly reduced the PCNA‐SIRF Mean Fluorescence Intensity (MFI)/nucleus in H460R but not H460S cells (Fig. 2F, left and right panels). Altogether, these results indicated that SPHINX31 decreases the amount of PCNA recruited at nascent replication forks in H460R cells. This could be consistent with enhanced replicative stress [55].

### 3.3 SPHINX31, as well as SRPK1 knock‐down, decrease ATR auto‐phosphorylation at Thr1989 and the recruitment of ATRIP and TOPBP1 proteins in chromatin‐enriched fractions

**Fig. 3:**
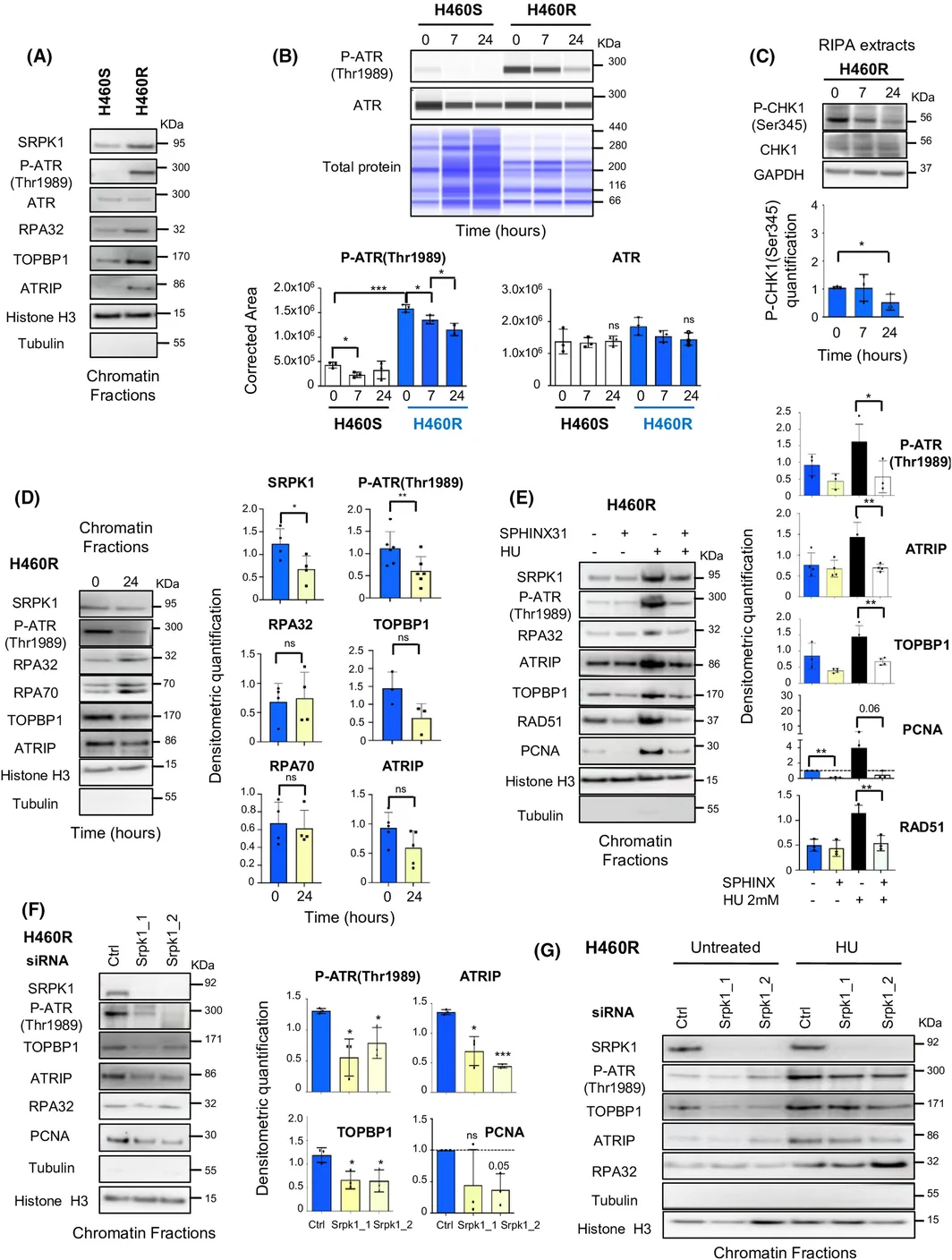
SPHINX31 inhibits ATR/CHK1 signaling pathway. (A) Representative immunoblots of indicated proteins in chromatin‐enriched fractions obtained from H460S and H460R cells *n* = 3. (B–D) H460S/R cells were treated or not (medium) with 10 μm SPHINX31 for the indicated times (hours) *n* = 3. (B) Upper panel: representative images of P‐ATR(Thr1989) and ATR proteins as detected by capillary western blot. Total protein was used as a control of equal loading. Lower panel: quantification (corrected area relative to total protein) of P‐ATR(Thr1989) (left) or total ATR (right) protein level. Mean ± SD *n* = 3. (C) Upper panel: representative immunoblots of P‐CHK1(Ser345) and CHK1 proteins in RIPA extracts. GAPDH was used as a loading control. Lower panel: densitometric quantification of P‐CHK1(Ser345) signal normalized to GAPDH signal. Mean ± SD *n* = 3. (D) Left panels: representative immunoblots of the indicated proteins in chromatin‐enriched fractions. Histone H3 and tubulin were used as controls of sub‐cellular fractionation. Right panels: densitometric quantification of specific signals normalized to histone H3 signal. Mean ± SD *n* = 3. (E) H460R cells were treated (+) or not (medium, −) for 24 h with 10 μm SPHINX31 in the presence or absence of 2 mm hydroxyurea (HU). Left panel: representative immunoblots of indicated proteins in chromatin‐enriched fractions. Histone H3 and tubulin were used as controls of subcellular fractionation. Right panel: densitometric quantification of each specific signal normalized to H3 signal. Mean ± SD *n* = 3. (F) H460R cells were transfected for 72 h with either control (Ctrl) siRNA or two distinct siRNAs targeting SRPK1 (Srpk1_1; Srpk1_2). Left panel: representative immunoblots of indicated proteins in chromatin‐enriched fractions. Right panel: densitometric quantification of P‐ATR(Thr1989), ATRIP, TOPBP1, and PCNA signals normalized to H3 signal. Mean ± SD *n* = 3. (G) H460R cells were transfected for 48 h with either control (Ctrl) siRNA or two distinct siRNAs targeting SRPK1 (Srpk1_1 and Srpk1_2) and treated or not for 24 additional hours with 2 mm hydroxyurea (HU). Representative immunoblots of indicated proteins in chromatin fractions Tubulin and histone H3 were us as controls of sub‐cellular fractionation *n* = 2. In all experiments, unpaired *t*‐test. ***P < 0.001, **P < 0.01, *P < 0.05, ns: not significant.

One of the main signaling pathways activated in response to replication stress depends on ATR [56, 57]. During replication stress, Replication Protein A (RPA) accumulates at stress‐induced single‐strand DNA (ssDNA) at forks and recruits the ATR‐ATRIP complex [58, 59]. ATR is then activated via interactions with either Ewing's Tumor‐Associated Antigen 1 (ETAA1) or TOPBP1 through their ATR Activation Domains (AADs) [60, 61]. In addition, recruitment of ATR‐ATRIP to RPA‐ssDNA leads to congregation of ATR‐ATRIP complexes and ATR auto‐phosphorylation at Thr1989 [62]. As shown in Fig. 3A, H460R cells displayed higher amount of P‐ATR(Thr1989), RPA32, ATRIP, and TOPBP1 proteins in chromatin‐enriched fractions in comparison to H460S cells. Accordingly, in a parallel study, we also showed by immunofluorescence that H460R cells exhibited more RPA32, γH2AX, and P‐53BP1(Ser1778) nuclear foci as compared to H460S cells (Jamal El Hussein et al., BioRxiv 2026.02.17.706284). These results could be consistent with H460R cells exhibiting a higher intrinsic level of replicative stress. This was not unexpected as activation of prosurvival replication stress pathways is a critical mechanism by which cancer cells escape to chemotherapeutic agents including cisplatin [63]. Capillary western blot confirmed that P‐ATR(Thr1989) accumulates in H460R compared to H460S cells (Fig. 3B). Importantly, SPHINX31 decreased levels of both activated P‐ATR(Thr1989) (Fig. 3B) and P‐CHK1(Ser345) (Fig. 3C) proteins in H460R cells, while it did not affect the total amount of ATR (Fig. 3B), ATRIP or TOPBP1 (Fig. S3A). Rather, SPHINX31 tended to decrease the recruitment of TOPBP1 and ATRIP proteins in chromatin‐enriched fractions (Fig. 3D). Although SPHINX31 did not affect SRPK1 total protein level (Fig. 1F), it decreased the amount of SRPK1 in chromatin‐enriched fractions (Fig. 3D). It was previously found that pharmacological inhibition of ATR using VE‐821 prevented the nuclear translocation of SRPK1 in 5‐fluorouracil‐treated HeLa and T24 cells [32]. In H460S or H460R cells, we did not observe a significant decrease of SRPK1 recruitment in chromatin‐enriched fractions upon VE‐821 treatment alone (Fig. S3B). Of note, inhibition of ATR auto‐phosphorylation by SPHINX31 in H460R cells was not a consequence of their accumulation in G_1_ phase of the cell cycle, as it started to occur after 7‐h treatment (Fig. 3B), before SPHINX31‐inhibited EdU incorporation (Fig. S1G). Ataxia Telangiectasia Mutated (ATM) has been shown to activate ATR in response to DNA double strand breaks (DSBs) [64, 65]. Therefore, we also tested the effects of SPHINX31 on ATM signaling. As shown in Fig. S3C,D, SPHINX31 did not significantly modulate P‐ATM(Ser1981) or P‐CHK2(Thr68) protein level. As compared to parental cells, higher basal levels of P‐ATM(Ser1981) and P‐CHK2(Thr68) were observed in resistant cells. Altogether, these data showed that SPHINX31 inhibits ATR/CHK1 signaling pathway in H460 resistant cells.

Hydroxyurea (HU) acts as an inhibitor of ribonucleotide reductase (RNR) that induces dNTP starvation and initially stalls replication forks, thereby inducing replicative stress [66, 67]. In order to extend our results, we analyzed the effects of SPHINX31 on HU‐induced ATR activation in H460R cells. SPHINX31 prevented HU‐induced accumulation of P‐ATR(Thr1989) and P‐CHK1(Ser345) (Fig. S3E), as well as the recruitment of ATRIP and TOPBP1 in chromatin‐enriched fractions (Fig. 3E). SRPIN340, another SRPK1 inhibitor [19, 20], also prevented HU‐induced accumulation of P‐ATR(Thr1989) and ATRIP recruitment in chromatin fractions although to a less extent (Fig. S3F). This could be explained by the fact that SRPIN340 exhibits lower activity and selectivity for SRPK1 over SRPK2 than that of SPHINX31 [21, 22]. As a whole, these data demonstrated that pharmacological inhibition of SRPK1 prevents the activation of ATR/CHK1 signaling pathway in response to replicative stress, highlighting SRPK1 as being part of this response. RAD 51 stabilizes stalled replication forks by mediating fork reversal and protection from breakage [68]. Moreover, PCNA was recently shown to protect DNA ends from enzymatic attack at stalled replication forks, thereby preventing their collapse [69]. Interestingly, we also observed that SPHINX31 decreases the amount of both proteins in chromatin‐enriched fractions following HU exposure (Fig. 3E). Such a decrease of PCNA upon SPHINX31 treatment in HU‐treated cells was confirmed by SIRF analysis (Fig. S3G).

Then, we tested whether the knock‐down of SRPK1 by using siRNA could recapitulate the effects of SPHINX31 in H460R cells. In whole cellular extracts, neutralization of SRPK1 decreased P‐ATR(Thr1989) and P‐CHK1(Ser345) protein levels which correlated with γH2AX accumulation, although this was mainly seen with one siRNA (Fig. S4A). More significantly, the knock‐down of SRPK1 decreased P‐ATR(Thr1989), ATRIP, TOPBP1, as well as PCNA protein levels in chromatin‐enriched fractions of H460R cells (Fig. 3F). Although less significant than SPHINX31, siRNAs against SRPK1 also tended to reduce the recruitment of TOPBP1 and ATRIP in chromatin‐enriched fractions of H460R cells treated with HU (Fig. 3G). As siRNA transfection does not equally knock‐down SRPK1 expression in all cells, this could explain this lower effect compared to SRPK1 pharmacological inhibition. In addition, SRPK1 knock‐down decreased P‐CHK1(Ser345) protein level in H460R cells treated with HU, which correlated with an increase of γH2AX protein level (Fig. S4B). Taken together, these results showed that neutralization of SRPK1 in H460R cells recapitulates to some extent the inhibitory effects of SPHINX31 on ATR signaling pathway. They reinforced the idea that SRPK1 might be a new actor of the replicative stress response.

Although SPHINX31 only slightly prevented cell growth of A549 parental and resistant cells (Fig. 1C), we investigated whether it impacts ATR signaling in these cells. As in H460R cells, a higher expression level of P‐ATR(Thr1989) was found in A549R compared to A549S cells (Fig. S5A) and SPHINX31 decreased P‐ATR(Thr1989) and ATR protein levels in whole cellular extracts of A549R cells (Fig. S5A,B). SPHINX31 also decreased ATRIP in both cell lines and TOPBP1 protein levels in A549R cells, an effect observed after 7‐h treatment (Fig. S5A,B). In addition, and despite high variability between biological replicates, SPHINX31 tended to decrease the recruitment of ATRIP and TOPBP1 in chromatin‐enriched fractions of A549S and A549R cells which was again more pronounced after 7‐h treatment as compared to 24‐h exposure (Fig. S5C). These results suggest that A549S/R cells might recover from SPHINX31‐induced ATR inhibition, explaining their lower sensitivity to SPHINX31‐mediated cytotoxic effects as compared to H460R cells.

### 3.4 SRPK1 is recruited at nascent replication forks upon replicative stress and favors the accumulation of TOPBP1 nuclear foci

**Fig. 4:**
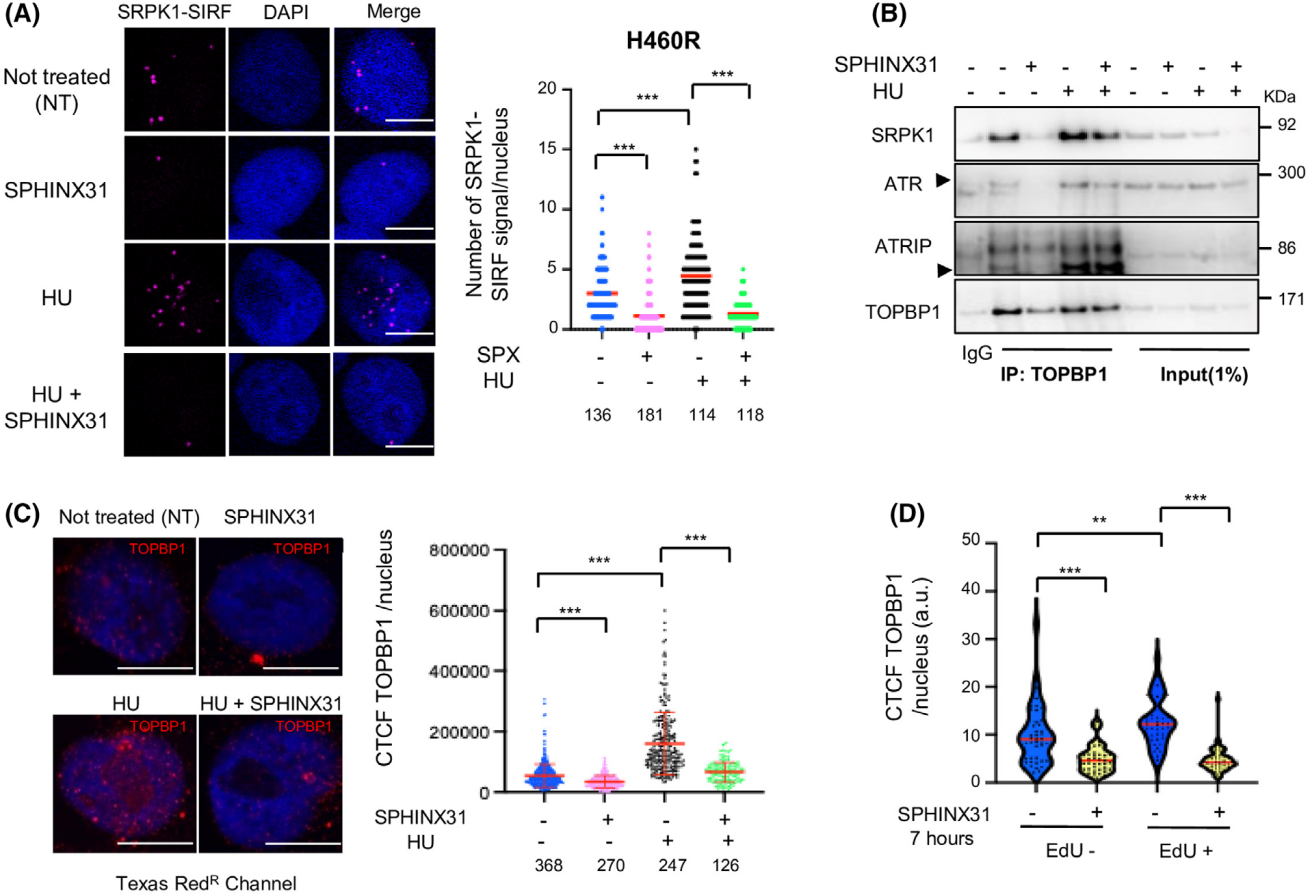
SRPK1 is recruited at nascent replication forks upon replicative stress and is a new component of the ATR/ATRIP/TOPBP1 complex. (A) Left panel: SRPK1‐SIRF signals in H460R cells treated or not (medium, NT) for 14 h with 5 μm SPHINX31. 2 mm hydroxyurea (HU) was added or not for 4 additional hours as indicated. Scale bar = 10 μm. Right panel: quantification of the number of SRPK1‐SIRF spots/nucleus. Mean ± SD *n* = 3. (B) H460R cells were treated (+) or not (medium, −) with 10 μm SPHINX31 in the presence or absence of 2 mm hydroxyurea for 24 h. Representative immunoblots of indicated proteins in TOPBP1 co‐immunoprecipitates extracted from chromatin fractions. IgG was used as a negative control. Input represents 1% of total immunoprecipitated proteins. (C) H460R cells were treated or not (medium, NT) for 24 h with 10 μm SPHINX31 in the presence or absence of 2 mm hydroxyurea as indicated. Left panel: representative images of TOPBP1 immunofluorescence staining. Scale bar = 10 μm. Right panel: TOPBP1 immunostaining quantification (corrected total cell fluorescence/nucleus, CTCF). Mean ± SD *n* = 3. (D) Quantification of TOPBP1 CTCF in H460R cells treated (+) or not (medium, −) for 7 h with 10 μM SPHINX31 according to either EdU‐positive or EdU‐negative cells. Mean is indicated in red *n* = 2. All unpaired *t*‐test, ***P < 0.001, **P < 0.01.

Having demonstrated that SPHINX31 as well as SRPK1 knock‐down negatively impacts ATR signaling in H460R cells (Fig. 3), we further deepened the molecular mechanisms by which SRPK1 could regulate ATR activation. First, we tested whether SRPK1 could be recruited at nascent replication forks by using SIRF. As shown in Fig. 4A, SRPK1‐SIRF spots were detected in nuclei of untreated H460R cells and their number decreased upon SPHINX31 treatment. Moreover, HU increased the number of SRPK1‐SIRF spots/nucleus and SPHINX31 reversed the effects of HU (Fig. 4A). Pulse‐chase experiments demonstrated that SRPK1‐SIRF signal was specific as it significantly decreased in thymidine treated cells (Fig. S6A). As a whole, these results provided the first evidence that SRPK1 can be recruited at stalled replication forks upon replicative stress and that SPHINX31 prevents this recruitment. These results led us to test whether SRPK1 could interact with the ATR‐ATRIP‐TOPBP1 complex. Consistently, in chromatin‐enriched fractions, we found that SRPK1 co‐immunoprecipitates with TOPBP1 together with ATR and ATRIP proteins (Fig. 4B). This interaction was slightly enhanced following HU exposure (Fig. 4B). The SRPK1/TOPBP1 interaction was confirmed by using Proximity Ligation Assay in H460R untreated and HU‐treated cells and SPHINX31 was found to decrease SRPK1/TOPBP1 PLA interaction (Fig. S6B). As TOPBP1 was previously found to form nuclear foci in cells exposed to HU [70, 71], we also performed immunofluorescence studies. TOPBP1 nuclear foci were detected in H460R cells (Fig. 4C). As expected, TOPBP1 nuclear intensity strongly increased upon exposure of these cells to HU (Fig. 4C). Interestingly, SPHINX31 significantly prevented TOPBP1 foci accumulation both in untreated and HU‐treated resistant cells (Fig. 4C), which was seen even after 7‐h treatment in both EdU‐positive and EdU‐negative cells (Fig. 4D). Altogether, these results demonstrated that SRPK1 is recruited at stalled replication forks upon replicative stress, co‐immunoprecipitates with TOPBP1/ATR/ATRIP complexes and contributes to TOPBP1 nuclear foci accumulation. These results unravel SRPK1 as a new component of the ATR/CHK1 replicative checkpoint.

### 3.5 SRPK1 directly interacts with TOPBP1 BRCT4/5 and BRCT7/8 domains in a phospho‐dependent and phospho‐independent manner

**Fig. 5:**
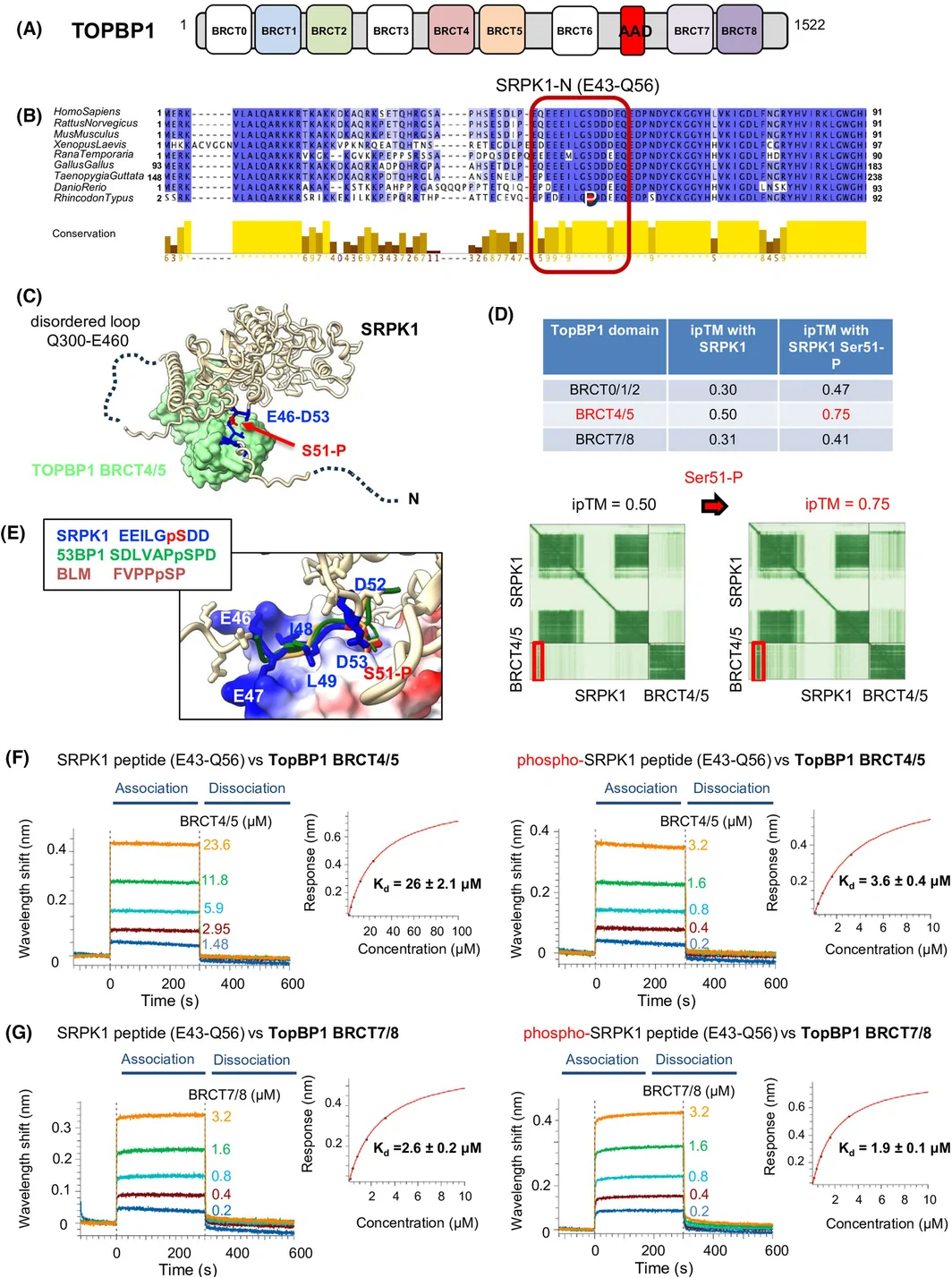
The SRPK1 N‐terminal region binds directly to the TOPBP1 BRCT4/5 and BRCT7/8 domains. (A) Schematic representation of TOPBP1 protein (1–1522) highlighting its 8 BRCT domains as well as its ATR Activation Domain (AAD). (B) Sequence alignment of the N‐terminal region of SRPK1. The region between residues 1 and 67 is predicted as disordered by AlphaFold. The folded catalytic domain begins around residue 68 (PBD references 5MXX and 5MY8). The sequence of the peptide used in the BioLayer Interferometry experiments (SRPK1‐N, panel E) is boxed in red. The serine 51 described as phosphorylated in a large number of proteomic studies is indicated by a P sign. (C) A model of the complex between SRPK1 and BRCT4/5 is presented. SRPK1 is in wheat, with its region between residues 43 and 56 at the interface with BRCT4/5 in blue. The side chains of this region are shown in sticks, with the phosphorylated serine 51 in red. TOPBP1 BRCT4/5 is in light green. (D) Upper panel: AlphaFold 3 (AF3) prediction of the interaction between the SRPK1 kinase and the TOPBP1 phosphopeptide binding domains BRCT0/1/2, BRCT4/5 and BRCT7/8. The table shows the AF3 interface scores obtained for SRPK1, either non phosphorylated or phosphorylated on Ser51, binding to the TOPBP1 domains BRCT0/1/2, BRCT4/5 and BRCT7/8. Lower panel: heat maps display the error on the residue relative position for the interaction between SRPK1 and BRCT4/5 (green—accurate versus white—inaccurate relative position). The region containing the information on an interaction between SRPK1‐N and BRCT4/5 is boxed in red. (E) Zoom on the interface between SRPK1 and BRCT4/5, showing how the SRPK1 region between residues 43 and 56 interacts with the TOPBP1 BRCT4/5 domain. Colors are as in (C). The AF3 model is superimposed onto the crystal structures of the 53BP1 (green; 6RMM) and BLM (orange; 5U6K) phosphorylated peptides bound to the TOPBP1 BRCT4/5 domain. (F, G) BioLayer Interferometry (BLI) experiments showing that SRPK1‐N, either non phosphorylated or phosphorylated at serine 51, interacts with a micromolar affinity with the BRCT4/5 (F) as well as the BRCT7/8 (G) domains of TOPBP1. Phosphorylation of serine 51 significantly increases binding to TOPBP1 BRCT4/5.

TOPBP1 features nine repetitions of a well‐folded protein–protein interaction motif, the BRCA1 C‐terminal domain (BRCT), and an ATR activation domain (AAD) [60, 72] (Fig. 5A). The TOPBP1 domains BRCT0/1/2, BRCT4/5, and BRCT7/8 have been shown to bind to phosphorylated peptides. Importantly, AlphaFold 3 (AF3) predicted that the N‐terminal disordered region of SRPK1 (Fig. 5B) interacts with the BRCT4/5 domain of TOPBP1 (aa 540–740) and that this interaction depends on phosphorylation of SRPK1 serine 51 (Fig. 5C,D). Indeed, the AF3 interface score (ipTM) increases from 0.50 (poorly significant) to 0.75 (robust prediction) upon phosphorylation of this serine. Several phospho‐proteomic studies identified the serine 51 phosphosite, and a previous publication reported that this site is phosphorylated by CK2 [73]. Comparison of our AlphaFold model with the crystal structures of the 53BP1 and BLM phosphorylated peptides bound to TOPBP1 BRCT4/5 revealed that the SRPK1‐N peptide is positioned as the 53BP1 and BLM peptides, with the side chain of the phosphorylated serine of SRPK1 being positioned in the cavity occupied by the phosphorylated residues of 53BP1 and BLM (Fig. 5E). In order to experimentally test whether the interaction between phosphorylated SRPK1 and TOPBP1 BRCT4/5 is direct, we performed a BioLayer Interferometry (BLI) experiment, in which we coated a biotinylated SRPK1‐N (E43‐Q56) peptide on a chip and incubated this chip in solutions of increasing amounts of purified BRCT4/5. We measured Kd values of 26 ± 2.1 μm and 3.6 ± 0.4 μm using SRPK1‐N either nonphosphorylated or phosphorylated at serine 51, respectively (Fig. 5F and Fig. S7A). This confirmed that the N‐terminal disordered region of SRPK1 is able to bind in a phospho‐dependent manner to TOPBP1 BRCT4/5. The same BLI experiment was performed with increasing amounts of purified BRCT7/8, which identified similar micromolar affinity for both the nonphosphorylated (measured *K*d value of 2.6 ± 0.2 μm) and the phosphorylated SRPK1 (measured *K*d value of 1.9 ± 0.1 μm) peptides (Fig. 5G and Fig. S7B). As a whole, these results demonstrated that SRPK1 directly interacts with TOPBP1 BRCT4/5 and BRCT7/8 domains in a phospho‐dependent and phospho‐independent manner, respectively.

Interestingly, upon examining the TOPBP1 protein sequence, we also noticed a stretch of 3 RS peptides within its AAD domain (1095–1127) that was reminiscent of RS‐enriched consensus sequences phosphorylated by SRPK kinases (Fig. S8A). We therefore performed an *‘in vitro’* kinase assay to test whether SRPK1 could phosphorylate TOPBP1's AAD. In this assay, SRPK1 phosphorylated its well‐known target, the N‐terminal part of lamin B receptor (LBRNt) between residues 62–92 [36], but it was not able to phosphorylate GST‐TOPBP1(978–1522), nor GST‐TOPBP1(978–1192) fragment that contain the residues 1095–1127 of AAD (Fig. S8A). The absence of phosphorylation by SRPK1 of a ^15^N labeled TOPBP1(1055–1177) fragment was confirmed by NMR (Fig. S8B). These results indicated that SRPK1 does not phosphorylate the TOPBP1's AAD in our *‘in vitro’* conditions.

### 3.6 SPHINX31 and SRPK1 regulate the splicing of *DBF4B* , *RFC5* and *WIZ*

**Fig. 6:**
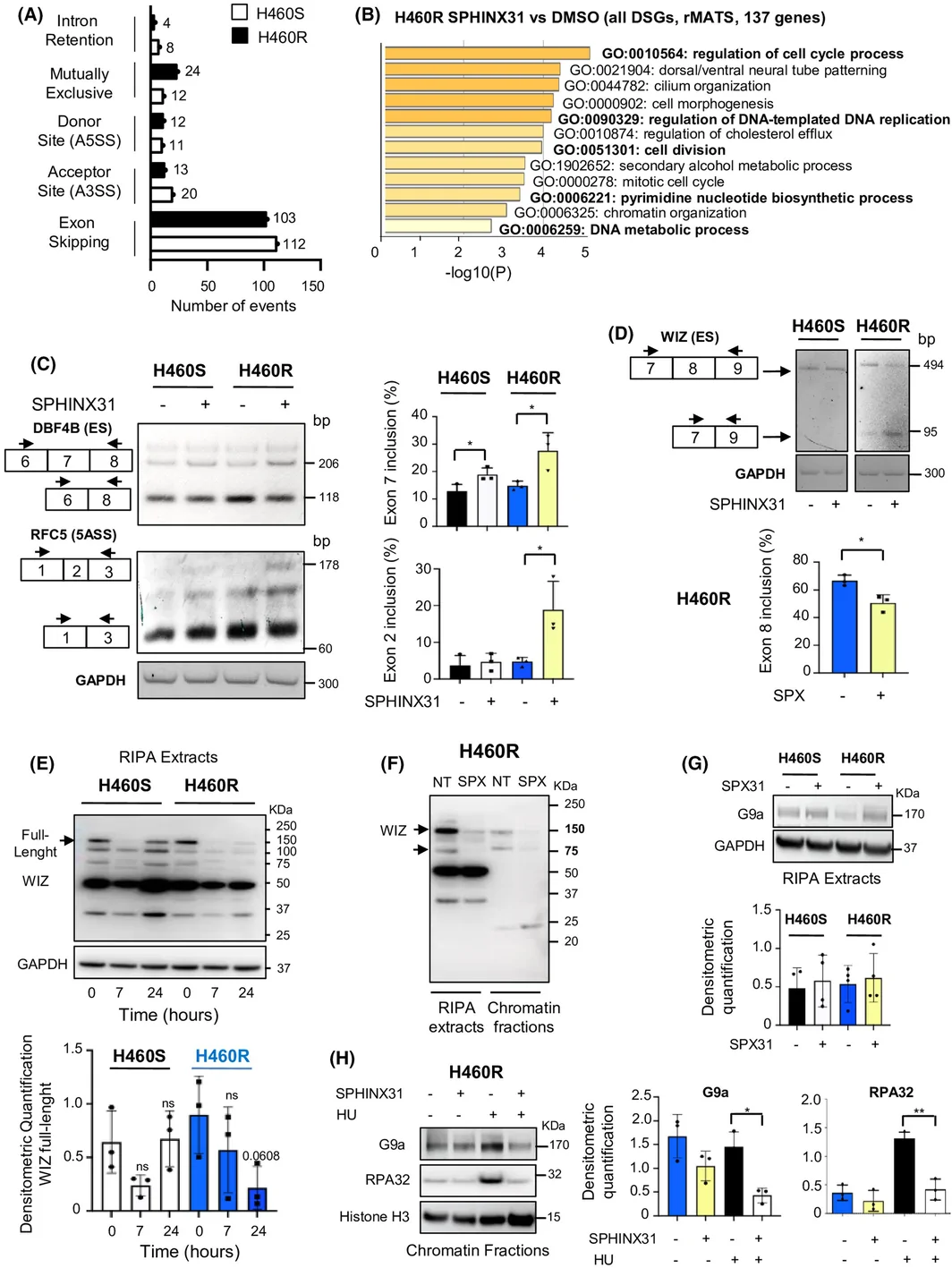
SPHINX31 regulates alternative splicing of *WIZ*, downregulates WIZ protein level, and decreases the amount of G9a protein in chromatin‐enriched fractions from HU‐treated cells. (A–D) H460S/R cells were treated for 24 h with 5 μm SPHINX31 or DMSO as a control. Comparison of Differentially Spliced Genes (DSGs) was done between SPHINX31‐ and DMSO‐treated conditions in H460S or H460R cells. (A) Number of differential alternative splicing events (DSGs), according to intron retention, mutually exclusive exons, donor site (5′ASS), acceptor site (3′ASS) or exon skipping. (B) Metascape analysis of all DSGs in H460R cells. (C, D) H460S and H460R cells were treated (+) or not (medium, −) for 24 h with 10 μM SPHINX31. RT‐PCR analyses were performed to study *DBF4B* (C, upper panels), *RFC5* (C, lower panels), or *WIZ* (D) splicing using specific primers (black arrows). (C, D) Left/upper panels: representative agarose gels of amplified products. *GAPDH* was used as an internal control of amplification. Numbers indicate amplicon size (in base pairs, bp). (C, D) Right/lower panels: densitometric quantification of exon inclusion (%). Mean ± SD *n* = 3. (E) H460 parental and resistant cells were treated or not (medium) with 10 μM SPHINX31 for indicated times. Upper panel: representative immunoblot of WIZ expression in RIPA extracts. GAPDH was used as a loading control. Lower panel: densitometric quantification (mean ± SD) of WIZ full‐length protein *n* = 3. (F) Representative immunoblot of WIZ in H460R treated (SPX) or not (medium, NT) with SPHINX31 for 24 h in RIPA extracts and chromatin‐enriched fractions as indicated. (G) H460 parental and resistant cells were treated or not (medium, NT) for 24 h with 10 μm SPHINX31. Upper panel: representative G9a immunoblot. GAPDH was used as a loading control. Lower panel: densitometric quantification (mean ± SD) *n* = 4. (H) H460R cells were treated (+) or not (medium, −) for 24 h with 10 μm SPHINX31 in the presence or absence of 2 mm hydroxyurea (HU) as indicated. Left panel: representative immunoblots of indicated proteins in chromatin‐enriched fractions. Histone H3 was used as a loading control. Right panel: densitometric quantification of G9a and RPA32 signals normalized to H3 signal. Mean ± SD *n* = 3. All unpaired *t*‐test **P < 0.01, *P < 0.05, ns: not significant.

The knock‐down or chemical inhibition of SRPK1, notably by using SPHINX31, has been shown to alter splicing in Wilms Tumor and melanoma as well as in Acute Myeloid Leukemia (AML) cells [23, 74, 75]. Therefore, beside analyzing the effects of SPHINX31 on ATR signaling pathway, we also performed RNA‐Seq experiments to identify genes differentially expressed (DEG) and/or spliced (DSG) upon SPHINX31 treatment in H460S and H460R cells (Tables S2–S4). To this end, H460S and H460R cells were treated with DMSO as a control or with 5 μm SPHINX31 for 24 h before RNA extraction and sequencing. As compared to untreated cells, 56 and 201 genes were differentially expressed in treated H460S and H460R cells, respectively (Fig. S9A; Table S2). Only 2 and 4 genes were both differentially expressed and spliced upon SPHINX31 treatment in H460S and H460R cells, respectively (Fig. S9B), indicating that SPHINX31 regulates distinct transcriptional and post‐transcriptional programs. Eight DSGs were commonly regulated by SPHINX31 in both cell lines (Fig. S9C). Exon skipping (ES) was the most prevalent alternative splicing event impacted by SPHINX31 (Fig. 6A). As quantified by rMATS, 112 and 103 ES events corresponding to 102 and 93 genes were differentially regulated by SPHINX31 in H460S and H460R cells, respectively. Interestingly, when Gene Ontology analysis was done on all DSGs using the Gene Annotation and Analysis Resources Metascape [51], GO‐terms related to the regulation of cell cycle process, regulation of DNA‐templated DNA replication and cell division were found to be enriched in H460R cells treated with SPHINX31 (Fig. 6B). Similar enrichment was found in H460S cells (Fig. S9D). In H460R cells, DSGs involved in DNA replication were DBF4B‐CDC7 Kinase Regulatory Subunit *(DBF4B)*, Replication Factor C Subunit 5 *(RFC5)*, Telomeric Repeat Binding Factor 1 *(TERF1)* and Widely Interspaced Zinc finger *(WIZ)*. Very interestingly, three of them encode proteins that play a role in ATR signaling pathway. DBF4B (also known as DRF1/ASKL1) is part of the DBF4/DBF4B‐dependent S‐phase promoting kinase Cdc7 which regulates initiation and progression of DNA replication [76, 77]. DBF4B has been shown to trigger DNA replication re‐initiation during S‐phase checkpoint recovery through regulation of ATR/CHK1 signaling [78]. RFC5 is a component of the RFC complex that binds to the 5′‐ended ssDNA‐dsDNA junctions at stalled replication forks and loads the 9‐1‐1 clamp complex which then recruits TOPBP1 with the help of RHINO and MRN complexes allowing stimulation of ATR [56]. WIZ interacts with the G9a‐GLP histone methyltransferase and regulates G9a protein stability [79], as well as facilitates the interaction of G9a with chromatin [80]. Importantly, G9a has been reported to directly interact with and to enhance chromatin recruitment of RPA to damage sites, which could participate in ATR/ATRIP recruitment and ATR activation [81]. RT‐PCR analyses were performed to validate RNA‐Seq data. The results showed that SPHINX31 promotes the inclusion of exon 7 of *DBF4B* both in H460S and H460R cells, and the inclusion of an alternative exon 2 of *RFC5* (selection of an alternative donor site) as well as the exclusion of exon 8 of *WIZ* in resistant cells (Fig. 6C and Fig. 6D). Similar results were obtained in H460R cells deprived of SRPK1 by siRNA for exon 7 inclusion of *DBF4B* (Fig. S9E) and exon 8 exclusion of *WIZ* (Fig. S9F). SPHINX31 also regulated *WIZ* splicing in A549 cells (Fig. S9G). Taken together, these results demonstrated that SPHINX31 regulates the alternative splicing of genes previously involved in ATR signaling pathway.

### 3.7 SPHINX31 decreases the expression of full‐length WIZ protein as well as the recruitment of G9a protein in chromatin‐enriched fractions

Up to now, nothing is known regarding the consequences of *WIZ* exon 8 skipping. In ENSEMBL, four *WIZ* transcripts that retain exon 8 have been described, namely *WIZ‐210* (ENST00000673675.1) which encodes the full‐length canonical WIZ protein, *WIZ‐211* (ENST00000674001.1), *WIZ‐214* (ENST00000864800.1), and *WIZ‐215* (ENST00000864804.1). Conversely, two additional *WIZ* transcripts with skipped exon 8 have been reported, namely *WIZ‐212* (ENST00000674089.4) and *WIZ‐202* (ENST00000389282.8). All these transcripts are predicted to encode proteins with four of them (WIZ‐214, WIZ‐215, WIZ‐212, WIZ‐202) exhibiting the same C‐terminal part than WIZ full‐length. By performing WIZ immunoblots in whole protein extracts using an antibody directed against this C‐terminal part, we first found that SPHINX31 decreases the level of full‐length WIZ protein (expected size 170KDa) (Fig. 6E). Using this antibody, additional bands migrating around 100KDa, 75KDa, 50KDa and 37KDa were also detected, with the 100KDa and 75KDa products tended to decrease upon treatment in H460R cells. Whether these bands correspond to translated products of alternative *WIZ* transcripts remains to be determined. Conversely, we did not observe any accumulation of isoforms that could be translated from *WIZ* transcripts devoid of exon 8 (*WIZ‐212*, *WIZ‐202*) in SPHINX31‐treated cells. Exon 8 of *WIZ* encodes 133 amino acids (residues 215–348) that are part of the second C_2_H_2_ zinc finger domain of WIZ (residues 224–246) that contributes to the binding of WIZ to DNA. Interestingly, the WIZ full‐length protein and the product migrating at 75KDa were the only ones detected in chromatin‐enriched fractions of H460R cells, and SPHINX31 strongly decreased their expression in these fractions (Fig. 6F). As previously mentioned, WIZ regulates the stability of G9a protein [79] and facilitates G9a interaction with chromatin [80]. Interestingly, G9a has been shown to enhance the chromatin recruitment of RPA to damage sites which could contribute to ATR activation [81]. Therefore, we tested the effects of SPHINX31 on G9a protein level in whole protein extracts and chromatin‐enriched fractions. A shown in Fig. 6G, in whole cellular extracts, SPHINX31 did not affect the total level of G9a protein in both H460S and H460R cells. In contrast, it decreased G9a protein level in chromatin fractions of HU‐treated H460R cells, that also correlated with decreased RPA recruitment (Fig. 6H). As a whole, these results showed that SPHINX31 decreases the level of WIZ protein as well as the level of chromatin‐bound G9a and RPA proteins in response to replicative stress, which could also account for ATR inhibition.

### 3.8 Combining SPHINX31 with DNA‐PKcs or CHK1 inhibitor leads to enhanced cell growth inhibition in NSCLC cells

**Fig. 7:**
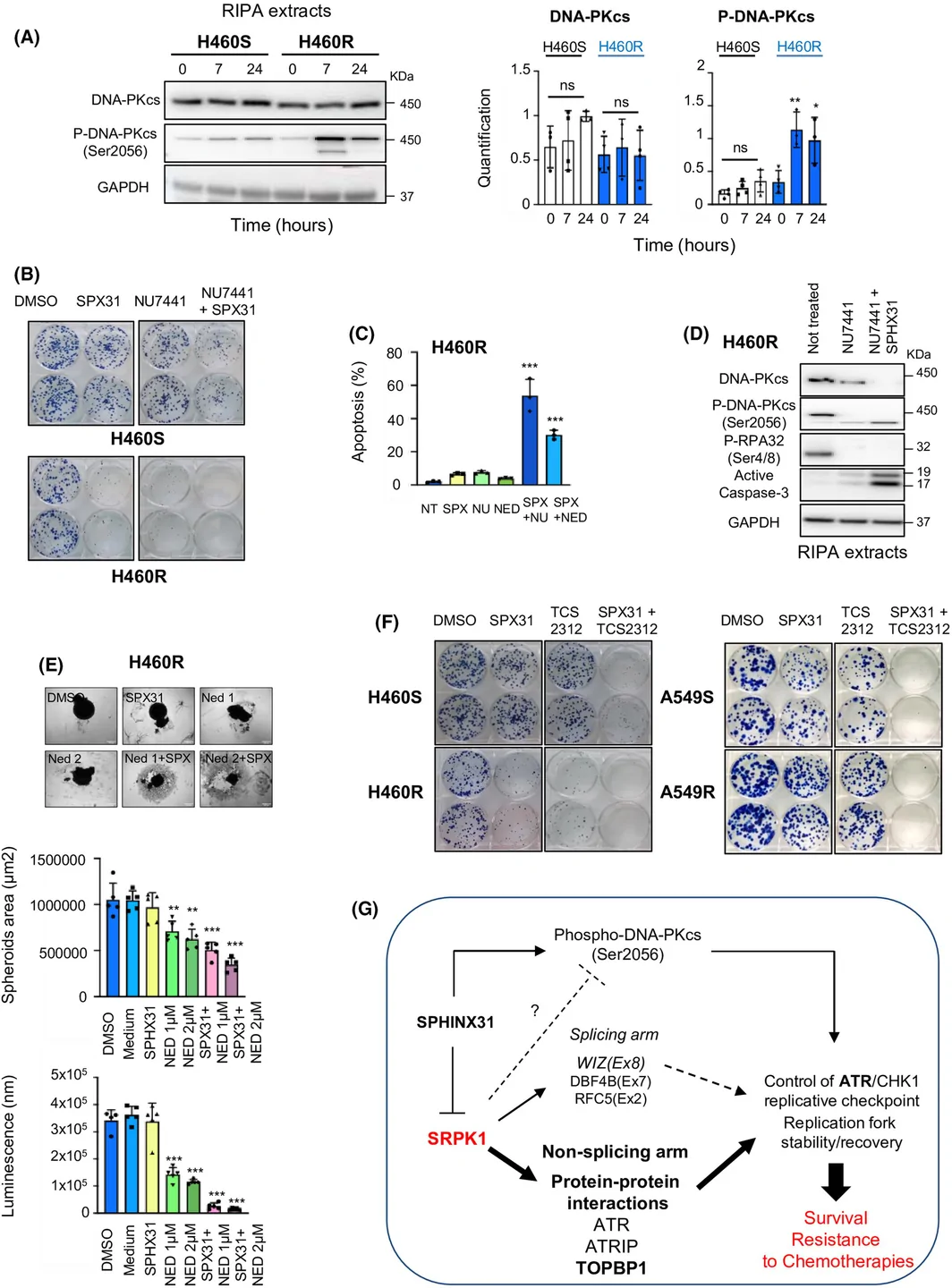
SPHINX31 exhibits enhanced cytotoxic effects when combined with DNA‐PKcs or CHK1 inhibitor in NSCLC cells. (A) H460S/R cells were treated or not (medium) for the indicated times with 10 μm SPHINX31. Left panel: representative immunoblot of indicated proteins. GAPDH was used as a loading control. Middle and right panels: densitometric quantification of DNA‐PKcs and P‐DNA‐PKcs(Ser2056) signals normalized to GAPDH signal. Mean ± SD *n* = 3. (B) Representative images of clonogenic assay in H460S and H460R cells treated or not (DMSO) for 14 days with 5 μm SPHINX31, 2 μm NU7441 or both *n* = 3. (C) Percentage of apoptosis detected by flow cytometry after active caspase‐3 staining in H460R cells treated or not (medium, NT) for 24 h with 10 μm SPHINX31, 5 μm NU7441, 2 μm nedisertib or a combination of SPHINX31 with each DNA‐PKcs inhibitor. Mean ± SD *n* = 3. (D) H460R cells were treated or not (medium) for 24 h with 5 μm NU7441 or a combination of 10 μm SPHINX31 with 5 μm NU7441. Representative immunoblot of indicated proteins. GAPDH was used as a loading control *n* = 3. (E) Spheroids derived from H460R cells were grown for 14 days in the presence or absence (DMSO, Medium) of 10 μm SPHINX31, 1 or 2 μm nedisertib or a combination of both drugs. Upper panel: representative images. Scale bar = 428 μm. Lower panels: measurement of spheroids area (μm^2^) and quantification of cell death by luminescence using CellTiter‐Glo^R^ 3D Cell Viability Assay. Mean ± SD. One experiment representative of 3 with 5 spheroids/condition. (F) Representative images of clonogenic assay in H460S/R and A549S/R cells treated or not (DMSO) for 14 days with 5 μm (H460S/R) or 10 μm (A549S/R) SPHINX31, or 1 μm TCS 2312 or both *n* = 3. All unpaired *t*‐test. *P < 0.05, **P < 0.01, ***P < 0.001, ns: not significant. (G) A working model of SRPK1 contribution to ATR/DNA‐PKcs/CHK1 replicative checkpoint. In NSCLC cells, SRPK1 is recruited at nascent replication forks upon replicative stress and interacts with ATR/ATRIP/TOPBP1 complex, in particular via TOPBP1 BRCT4/5 and BRCT7/8 domains. By this way, through splicing‐independent functions, SRPK1 participates to the replicative stress response and the full activation of the ATR/CHK1 checkpoint, and contributes to replication fork stability/recovery, maintenance of genomic stability and ultimately cell survival. This could also support a role of SRPK1 in resistance to chemotherapies. In addition, and although this remains to be further clarified, SRPK1 regulates alternative splicing of *WIZ* in favor of splice variants retaining exon 8, including that encoding the full‐length WIZ protein. WIZ contributes to the recruitment of G9a and the loading of RPA at stalled replication forks, which likely participates in ATR activation also. By targeting SRPK1, SPHINX31 inhibits ATR signaling pathway and leads to genomic instability notably in NSCLC cells with acquired resistance to platinum salts that exhibit high intrinsic level of replicative stress. Conversely, SPHINX31 activates DNA‐PKcs signaling that counteracts defective ATR thereby supporting the interest to combine SRPK1 inhibitor with DNA‐PKcs or CHK1 inhibitor to increase cytotoxic effects.

Our results demonstrated so far that SPHINX31 acts as an ATR inhibitor (ATRi). Although SPHINX31 treatment was associated with a clear inhibition of cell growth in H460R cells, its apoptotic effects, although significant, remained modest (Fig. 1E). It has been previously shown that, in ATRi‐treated cells escaping cell death, ATRi triggered a DNA‐PKcs and CHK1‐mediated back‐up pathway that creates a threshold of tolerable replication stress allowing cells to survive [82]. Therefore, we investigated the effects of SPHINX31 on DNA‐PKcs. In both parental and resistant cells, SPHINX31 did not modify DNA‐PKcs protein level (Fig. 7A). Rather, it activated DNA‐PKcs as detected by the accumulation of phospho‐DNA‐PKcs(Ser2056), mostly in resistant cells (Fig. 7A). Importantly, combining SPHINX31 with NU7441, a selective inhibitor of DNA‐PKcs, led to a stronger decrease of colony formation compared to each treatment alone in H460S and H460R cells, although this was less evident in H460R cells that already displayed higher sensitivity to each treatment alone (Fig. 7B). Apoptosis was also strongly increased in H460R cells treated with a combo SPHINX31/NU7441 or SPHINX31/nedisertib, another selective DNA‐PKcs inhibitor, compared to each treatment alone (Fig. 7C). In these cells, a cotreatment combining NU7441 + SPHINX31 induced P‐DNA‐PKcs(Ser2056) cleavage, decreased protein levels of DNA‐PKcs and its target phospho‐RPA32(Ser4/8), and led to the accumulation of active caspase‐3 (Fig. 7D). The reduced level of DNA‐PKcs upon combinatory treatment could be related to enhanced apoptosis as DNA‐PKcs is cleaved by caspase‐3 during apoptosis [83]. The enhanced cytotoxicity when combining SPHINX31 and DNA‐PKcs inhibition was confirmed in 3D culture spheroids selecting nedisertib, as it is a more potent selective inhibitor of DNA‐PKcs compared to NU7441 [84] (Fig. 7E). CHK1 is the final common target of ATR/DNA‐PKcs signaling pathways. Therefore, we tested the effects of combining SPHINX31 with TCS 2312, a CHK1 inhibitor. The results of clonogenic assays showed that this combination was even more efficient than the combo SPHINX31/DNA‐PKcs inhibitor whatever the NSCLC cells, including A549S/R cells that were moderately sensitive to SPHINX31 alone (Fig. 7F). Taken together, these results identified potent therapeutic strategies combining SPHINX31 with DNA‐PKcs or CHK1 inhibitor in NSCLC cells, including those with acquired resistance to platinum salts.

### 3.9 *SRPK1* mRNA level correlates with that of *PRKDC, TOPBP1,* *ATR* , *RFC5* or *DBF4B* in LUAD patients

Our results demonstrating a link between SRPK1 and the replicative stress response in NSCLC cells led us to look at potential relationships between SRPK1 and components of ATR/DNA‐PKcs signaling in LUAD patients. We first took advantage of public transcriptomic data from TCGA. We showed that *SRPK1* mRNA level positively correlates with that of *ATR* (Pearson *r* = 0.3759, *P* < 0.0001), *TOPBP1* (Pearson *r* = 0.4599, *P* < 0.0001), *PRKDC* encoding DNA‐PKCs (Pearson *r* = 0.5266, *P* < 0.0001), *RFC5* (Pearson *r* = 0.3498, *P* < 0.0001) or *DBF4B* (Pearson *r* = 0.1091, *P* = 0.0119) (Fig. S10A). As a comparison, the Pearson correlation between *ATR* and *TOPBP1*, *PRKDC*, *RFC5*, or *DBF4B* mRNA levels were 0.5873, 0.4839, 0.1927, and 0.09149, respectively (Fig. S10B). Moreover, looking for correlations between SRPK1 and either DNA‐PKcs, ATR, ATRIP, or TOPBP1 protein levels among 77 lung cancer cell lines, using quantitative proteomics of the Cancer Cell Line Encyclopedia database, we found a positive correlation between SRPK1 and ATR protein levels (*r* = 0.2902, *P* = 0.0121; Fig. S10C). Although we cannot conclude on direct protein–protein interaction, nor functional relationships in these analyses, these correlative results suggest a link between SRPK1 and components of the ATR/DNA‐PKcs signaling pathways in NSCLC patients.

## 4 Discussion

Spliceosome inhibitors represent a new class of promising anticancer drugs. However, their molecular mechanisms of action remain largely unknown, notably in lung cancer. In this study, we show that SPHINX31, which targets the kinase SRPK1, inhibits the ATR/CHK1 signaling pathway and cell proliferation in NSCLC cells, including cellular models with acquired resistance to platinum salts. At the molecular level, we highlight SRPK1 as a new component of the ATR/ATRIP/TOPBP1 replicative stress checkpoint. We show that SRPK1 is recruited at replication forks upon replicative stress and promotes ATRIP and TOPBP1 recruitment at chromatin as well as TOPBP1 nuclear foci accumulation. Importantly, SRPK1 is a novel direct interactor of TOPBP1 BRCT4/5 and BRCT7/8 domains. Concomitantly, we show that SRPK1 regulates the splicing of genes involved in DNA replication, such as *WIZ*, a gene previously linked to replicative stress [39, 40, 41]. We propose that the contribution of SRPK1 to the management of replicative stress response and the maintenance of genome stability participates to lung cancer cells survival and resistance to chemotherapies in NSCLC (Fig. 7G). Interestingly, in a parallel study (Jamal El Hussein et al., bioRxiv 2026.02.17.706284), we also found that pladienolide B, an inhibitor of SF3B1, a core component of the spliceosome, induces transcription‐dependent replicative stress in NSCLC resistant cells as well as massively downregulates the transcription and/or splicing of genes involved in DNA damage signaling and repair, including *ATR* and *PRKDC*. Therefore, although distinct mechanisms, targeting various components of the splicing machinery could provide a therapeutic benefit in NSCLC, including patients who escape chemotherapy, through inhibition of the DNA damage response.

In cancer cells, SRPK1 shuttles between the cytoplasm and the nucleus, a function primarily ascribed to the regulation of splicing through the control of SR proteins phosphorylation [85, 86]. However, several studies have now reported nuclear translocation of SRPK1 in response to cellular stress [87, 88], including genotoxic stresses [31, 32, 37, 38], supporting a role of nuclear SRPK1 during the DNA damage response that could also involve splicing‐independent‐functions [37]. In this study, we demonstrated that the amount of chromatin‐bound SRPK1 increases in lung cancer cells with acquired resistance to platinum salts as compared to parental cells (Fig. 3A). Similar more prominent SRPK1 nuclear staining was observed in breast cancer cells with secondary resistance to cisplatin [31]. ATR was previously shown to phosphorylate SRPK1 in response to 5‐fluorouracil leading to SRPK1 nuclear localization in HeLa and T24 cells [32]. In H460 resistant cells, inhibiting ATR with VE‐821 did not significantly impact the amount of chromatin‐bound SRPK1, although these cells exhibit high basal level of activated, auto‐phosphorylated ATR. Therefore, ATR‐mediated control of SRPK1 sub‐cellular distribution could vary depending on the cellular context. We further uncovered that SRPK1 is recruited at nascent replication forks in NSCLC resistant cells (Fig. 4A). SRPK1 was not detected in two previous large‐scale studies that used iPOND coupled with mass spectrometry in HEK293T cells [39, 40]. As HEK293T cells are noncancerous cells far from lung cancer cells, this could explain why SRPK1 is absent from these datasets. However, in Wessel et al. [89], an iPOND‐SILAC analysis was done in replicating cells which included two cancer cell lines, namely HCT116 and HeLa. Again, SRPK1 was not in the list of the 593 proteins found to be enriched at replication forks and nascent chromatin. One explanation could rely on the low‐abundance recruitment of SRPK1, precluding its detection in this study. Consistent with this hypothesis, another study using nascent chromatin capture to profile chromatin proteome dynamics during replication in HeLa cells provided a list of 3.995 proteins which included SRPK1, although at a low abundance [90]. Alternatively, SRPK1 could be mainly part of the replication stress proteome at stalled replication forks. Hence, in H460R cells, we showed that SRPK1 detection at replication forks is enhanced after treatment with hydroxyurea (Fig. 4A). The slight detection of SRPK1 at replication forks in nontreated H460R cells that exhibit enhanced basal replicative stress is also consistent with such a possibility.

What could be the biological consequences of SRPK1 recruitment at stalled replication forks? We provided further evidence that SPHINX31 or SRPK1 knock‐down decreases ATR(Thr1989) auto‐phosphorylation and downstream signaling in NSCLC cells. In response to replicative stress, the recruitment of ATR‐ATRIP to RPA‐ssDNA leads to congregation of ATR‐ATRIP complexes and ATR auto‐phosphorylation at Thr1989 [62]. Once phosphorylated on Thr1989, ATR is recognized by TOPBP1 [60]. BRCT4/5 and 7/8 domains of TOPBP1 have been shown to play a critical role for TOPBP1's function in ATR signaling [91]. Hence, TOPBP1's BRCT domains 7 and 8 enable TOPBP1 to engage ATR/ATRIP, to stimulate the ATR kinase, and to facilitate ATR substrate recognition [62]. Moreover, TOPBP1 BRCT4/5 domains function downstream of TOPBP1 recruitment to promote ATR‐mediated phosphorylation of CHK1 [91]. In parallel, phosphorylation of TOPBP1 inside its ATR Activation domain (AAD; residues 978–1192) controls its accumulation inside nuclear foci [70, 71], as well as its assembly into biomolecular condensates that act as feedback amplification mechanism to increase ATR signaling [92]. Importantly, we further demonstrated that SRPK1 co‐immunoprecipitates with ATR/ATRIP/TOPBP1 complexes, directly interacts with TOPBP1 BRCT4/5 and BRCT7/8 domains, which is dependent on SRPK1 phosphorylation on Ser51 residue for BRCT4/5 binding, and contributes to the recruitment of ATRIP and TOPBP1 on chromatin as well as to the accumulation of TOPBP1 into nuclear foci in response to hydroxyurea. These results unravel a new nuclear function of SRPK1 as a direct partner of the ATR/ATRIP/TOPBP1 complex which could contribute to ATR full activation. In addition, they add SRPK1 to the list of previously known interactors of TOPBP1's BRCT 4/5 region, namely 53BP1 [93] and the Bloom Syndrome Protein (BLM) helicase [94]. Despite identifying serine‐rich arginine (SR) sequences inside the AAD domain of TOPBP1, we did not succeed in showing SRPK1‐mediated phosphorylation of TOPBP1 *‘in vitro’*. However, as TOPBP1 functions as an oligomer which can simultaneously bind distinct partners and TOPBP1's AAD is active as a tetramer [95], our results do not exclude the possibility that SRPK1 phosphorylates oligomeric form of TOPBP1 in cellular context, which might also require additional binding partners on TOPBP1.

Once activated at stressed replication forks, ATR orchestrates a multifaceted response that protects the integrity of the genome and allows cell survival ([56], for review). In this setting, ATR functions include cell cycle arrest, stabilization of replication forks, and/or promotion of fork repair and restart ([56, 57], for reviews). In SPHINX31‐treated cells, SIRF experiments showed decreased recruitment of PCNA at nascent replication forks (Fig. 2E), consistent with fork stalling [40, 55, 96]. In response to replicative stress, stalled fork protection needs to stabilize a RAD51‐ssDNA filament to prevent nuclease action, thereby protecting reversed replication forks from collapse [97]. Moreover, it was recently shown that ATR/CHK1 prevents accumulation of excess Okazaki fragments that deplete PCNA from replication forks and lead to deprotection of DNA ends, enzymatic attack and fork collapse [69]. Importantly, SPHINX31 decreased P‐ATR(Thr1989) accumulation as well as the level of chromatin‐bound PCNA and RAD51 proteins in HU‐treated cells (Fig. 3E). Therefore, it is tempting to speculate that SPHINX31 might ultimately lead to replication fork collapse.

Until now, only a few genes which splicing regulation supports the anti‐tumoral properties of SRPK1 inhibitors have been identified in cancer cells. They include *VEGF‐A* in favor of anti‐angiogenic splice variants in prostate cancer cells [98, 99], cervical carcinoma and melanoma cells [100], or *BRD4* in favor of the anti‐proliferative splice variant in leukemic cells [75]. In this study, we identified additional genes which splicing is regulated by SPHINX31 or SRPK1 knock‐down in NSCLC cells. Interestingly, SPHINX31‐regulated spliced events were found to be enriched in genes involved in the regulation of cell cycle and DNA replication, such as *CDCA3*, *WIZ*, *DBF4B*, *RFC5*, or *TERF1*. Up to now, the consequences of alternative splicing of these genes are poorly known. We identified the skipping of exon 8 of *WIZ* as a splicing event regulated by SPHINX31 as well as SRPK1 knock‐down. Further, we demonstrated that SPHINX31 decreases the expression of the WIZ full‐length protein, which is encoded by a transcript retaining exon 8 and can be detected in chromatin‐enriched fractions from H460 resistant cells (Fig. 6F). Several observations argue for a link between WIZ and replicative stress. WIZ overexpression promotes resistance to drugs inhibiting DNA replication [101]. The knock‐down of WIZ in U2OS osteosarcoma cells leads to a significant downregulation of fork speed [39]. The knock‐down of ZNF644, which has a high degree of sequence identity with WIZ, also results in decreased proliferation, hypersensitivity to replication stress, and an increased amount of DNA damage in replicating cells [40]. WIZ also interacts with the G9a‐GLP histone methyltransferase and regulates G9a and GLP protein stability [79], as well as facilitates the interaction of G9a with chromatin [80]. G9a directly interacts with and enhances chromatin recruitment of RPA to damage sites, which could contribute to ATR/ATRIP recruitment and ATR activation [81]. Importantly, we further demonstrated that SPHINX31 decreases the amount of G9a loaded on chromatin in cells treated with hydroxyurea, which correlated with the decrease of RPA loading (Fig. 6H). Therefore, and although this remains speculative, all in all these results highly suggest that SPHINX31, by downregulating the level of chromatin‐bound WIZ protein, prevents the recruitment of G9a and the loading of RPA in response to replicative stress, and by the way decreases activation of ATR. As G9a/GLP complex recruited at replication forks is needed to prevent replication‐associated DNA damage [40], deciphering whether decreased G9a recruitment upon SPHINX31 treatment in HU‐treated cells also contributes to fork collapse requires further investigations. Moreover, whether and how the products derived from additional *WIZ* transcripts retaining or not exon 8 regulate this process remains to be determined.

We finally showed that SPHINX31, although inhibiting ATR, concomitantly activates DNA‐PKcs in resistant NSCLC cells. This is reminiscent of a previous study demonstrating DNA‐PKcs activation in cells displaying moderate replication stress upon treatment with ATR inhibitors [82]. Therefore, the activation of DNA‐PKcs upon SPHINX31 treatment could lower its overall cytotoxicity. Consistently, we demonstrated that combining SPHINX31 with NU7441 or nedisertib, two potent DNA‐PKcs inhibitors, significantly increases cellular apoptosis in both parental and resistant H460 cells. Furthermore, the combination of SPHINX31 with a CHK1 inhibitor was even more potent in inhibiting the cell growth of H460 and A549 parental and resistant NSCLC cells. In a murine AML model, synergistic effects without noticeable toxicity were previously found by combining SPHINX31 and i‐BET‐151, an epigenetic drug targeting BRD4 [75]. As BRD4 inhibition sensitized cancer cells to various replication stress‐inducing agents and synergized with ATR inhibitor [102], it remains to be determined whether SPHINX31 effects in AML also rely on inhibition of the ATR/CHK1 replicative checkpoint. We propose that combining SRPK1 inhibitor together with DNA‐PKcs or CHK1 inhibitor could provide additional therapeutic benefit in some NSCLC patients, including those who relapse after platinum salts‐based chemotherapy.

## 5 Conclusions

In this study, we unraveled a new nuclear function of the kinase SRPK1 in NSCLC cells, more particularly in cells with secondary resistance to platinum salts. Mechanistically, we demonstrated that SRPK1 directly binds to TOPBP1 and is a new component of the ATR/DNA‐PKcs/CHK1 replicative checkpoint. We further showed that SRPK1 controls the splicing of genes previously related to ATR signaling, such as *WIZ*. We propose that the contribution of SRPK1 to the management of replicative stress response and the maintenance of genome stability, through both splicing‐independent and ‐dependent functions, participates in lung cancer cells' survival and resistance to chemotherapies in NSCLC. Therefore, pharmacological inhibitors targeting SRPK1, such as SPHINX31, could provide a therapeutic benefit in NSCLC patients, notably those who escape chemotherapy.

## Conflict of interest

The authors declare no conflict of interest.

## Author contributions

BE designed the study, supervised the experiments, and wrote the manuscript. AS performed the majority of the experiments in H460 and CAL33 parental and resistant cells, analyzed and quantified the results with BE, prepared synthetic graphs, and wrote the methods section. MR performed SIRF analyses of PCNA. NZ did experiments in A549S and R cells. AG and CO provided technical support to AS and NZ for some biological, molecular, and biochemical experiments. AG, PS, TZ, and FD performed the immunofluorescence (AG, PS, TZ), immunoblotting (AG, PS, TZ, FD), and active caspase‐3 flow cytometry (FD) experiments required during the revision of the manuscript. HP and DA performed bioinformatic analyses of RNA‐Seq. AMC and CG did and analyzed NanoTemper Monolith experiments which were not included in the final version. HZ, SM, and SZJ performed AlphaFold3 predictions, Biolayer Interferometry and NMR experiments. EN performed and analyzed SRPK1 *‘in vitro’* kinase assay. TJ analyzed the TCGA LUAD cohort as well as did correlative analyses based on CCLE database. BE acquired fundings and edited the manuscript. All authors reviewed and approved the final version of the manuscript.

## Supporting information


**Fig. S1.** SPHINX31 decreases nuclear level of SRPK1 and increases 53BP1 nuclear foci in H460 resistant cells. (A) MTS assay in H460S and H460R cells treated or not (medium) for 96 h with increasing concentrations of cisplatin (μm). Mean ± SD *n* = 3. (B) A representative western‐blot of active caspase‐3 in H460S/R cells treated with 50 μm cisplatin for the indicated times. GAPDH was used as a loading control *n* = 3. (C) MTS viability assay in A549 parental and resistant cells treated or not (medium) for 72 h with increasing concentrations of cisplatin. Mean ± SD *n* = 3. (D) A 14 days clonogenic assay in A549 parental and resistant cells treated or not (DMF) with 1 μm cisplatin. (E) Left panels: representative SRPK1 immunostainings of H460S (a, b) and H460R (c, d) cells, treated (b, d) or not (DMSO; a, c) with 10 μm SPHINX31 for 24 h. Nuclei are outlined in yellow. Scale bar = 20 μm. Right panel: histogram quantifying SRPK1 nuclear intensity expressed as fold change relative to the DMSO condition. Data are shown as mean ± SD from 2 independent experiments. (F) Upper panels: representative immunofluorescence of EdU‐Cy5 staining. DAPI was used to counterstain the nucleus. Scale bar = 10 μm. Lower panel: quantification of the percentage of EdU‐positive cells. Each point represents the % of EdU‐positive cells in at least 10 nuclei/field. Untreated (medium, NT). Mean ± SD. *n* = 2 (> 50 nuclei counted/experiment). (G) H460R cells were treated (SPX) or not (DMSO) with 10 μm SPHINX31 for indicated times (hours). Upper panel: representative immunofluorescence staining of 53BP1. DAPI was used to counterstain the nucleus. Scale bar = 3 μm. Lower panel: quantification of the percentage of cells with ≥ 1 53BP1 nuclear foci. Mean ± SD *n* = 3. Unpaired *t*‐test. *P < 0.05, **P < 0.01.

**Fig. S2.** Controls of PCNA‐SIRF analysis. (A) H460S/R cells were treated or not (medium) with 10 μm SPHINX31 for the indicated times. Left panel: PCNA representative immunoblot. GAPDH was used as a loading control. Right panel: densitometric quantification of PCNA signal normalized to GAPDH signal. Mean ± SD *n* = 3. Unpaired *t*‐test. ns: not significant. (B) Representative illustrations of PCNA‐SIRF controls including staining of EdU‐positive cells with AlexaFluor‐488 azide and SIRF signals that were observed with no antibody, anti‐biotin antibody alone, anti‐PCNA antibody alone, or both anti‐biotin and anti‐PCNA antibodies. Scale bar = 10 μm. (C) PCNA/biotin‐SIRF was done with or without clicking EdU to biotin. In the absence of click‐it reaction, no SIRF signal can be detected. (D) After 10 min pulse with EdU, pulse‐chase was done by incubating H460R cells with 10 μm thymidine (Thym) for 2 h before doing PLA PCNA/Biotin. PCNA‐SIRF signal strongly decreased in thymidine‐treated cells. Unpaired *t*‐test. ****P < 0.0001.

**Fig. S3.** SPHINX31 inhibits ATR/CHK1 but not ATM/CHK2 signaling. (A) H460S/R cells were treated or not (medium) with 10 μm SPHINX31 for the indicated times (hours). Representative immunoblots of indicated proteins in RIPA extracts *n* = 3. (B) H460S/R cells were treated or not (medium) with 10 μm VE‐821 for the indicated times (hours). Upper panels: representative immunoblots of SRPK1 in chromatin‐enriched fractions. Histone H3 and tubulin were used as controls of sub‐cellular fractionation. Lower panel: SRPK1 densitometric quantification. Mean ± SD *n* = 3. (C, D) H460S/R cells were treated or not (medium) with 10 μm SPHINX31 for the indicated times (hours). (C) Representative immunoblots of indicated proteins in RIPA extracts. (D) P‐ATM(Ser1981)/ATM ratio and P‐CHK2(Thr68) densitometric quantifications. Mean ± SD *n* = 3–4. (E) H460R cells were treated (+) or not (medium, −) for 24 h with 10 μm SPHINX31 in the presence or absence of 2 mm hydroxyurea (HU). Left panel: representative immunoblots of the indicated proteins in whole protein extracts. Right panels: densitometric quantification. Mean ± SD *n* = 4–5. (F) H460R cells were treated (+) or not (medium, −) for 24 h with 40 μm SRPIN340 in the presence or absence of 2 mm hydroxyurea (HU). Upper panel: Representative immunoblots of indicated proteins in chromatin‐enriched fractions. Histone H3 and tubulin were used as controls of sub‐cellular fractionation. Lower panel: densitometric quantification of the indicated proteins. Mean ± SD *n* = 2–3. (G) Hydroxyurea (2 mm) was added for 4 h on H460R cells having been pre‐treated or not with 5 μm SPHINX31 for 14 h. Upper panels: representative images of PCNA‐SIRF, EdU and DAPI signals. Scale bar = 10 μm. Lower panel: quantification of PCNA‐SIRF fluorescence intensity/nucleus. Mean. *n* = 2. All, unpaired *t*‐test. ****P < 0.0001, **P < 0.01, *P < 0.05, ns: not significant.

**Fig. S4.** SRPK1 knock‐down downregulates ATR/CHK1 signaling in H460 resistant cells. (A) H460R cells were transfected for 72 h with either control (Ctrl) siRNA or two distinct siRNAs targeting SRPK1 (Srpk1_1 and Srpk1_2). Left panel: representative immunoblots of indicated proteins in whole cellular extracts. Right panels: densitometric quantification of specific signal according to GAPDH. Mean ± SD *n* = 3. (B) H460R cells were transfected for 48 h with either control (Ctrl) siRNA or two distinct siRNAs targeting SRPK1 (Srpk1_1 and Srpk1_2) and treated or not (medium) for 24 additional hours with 2 mm hydroxyurea (HU). Upper panels: representative immunoblots of indicated proteins in RIPA extracts. GAPDH was used as a loading control. Lower panels: densitometric quantification of P‐CHK1(Ser345) or γH2AX protein normalized to GAPDH used as a loading control. Mean ± SD *n* = 3–4. All, unpaired *t*‐test **P < 0.01, *P < 0.05.

**Fig. S5.** SPHINX31 inhibits ATR/CHK1 signaling in A549 parental and resistant NSCLC cells. (A–C) A549S and A549R cells were treated or not (medium) with 10 μm SPHINX31 for indicated times. (A) Left panels: representative immunoblots of the indicated proteins in RIPA extracts. GAPDH was used as a loading control. Right panels: densitometric quantification of P‐ATR(Thr1989) signals normalized to GAPDH. Mean ± SD *n* = 3. (B) Densitometric quantifications (fold change) of SRPK1, ATRIP, ATR, or TOPBP1 signals obtained from (A) and normalized to GAPDH. (C) Left panels: representative immunoblots of the indicated proteins in chromatin‐enriched fractions. Histone H3 was used as a loading control. Right panel: densitometric quantification (fold change) of indicated proteins. Mean ± SD *n* = 3–4. All, unpaired *t*‐test. **P < 0.01, *P < 0.05, ns: not significant.

**Fig. S6.** SRPK1 is recruited at nascent replication forks and is in close proximity with TOPBP1 in response to replicative stress. (A) Negative control of SRPK1‐SIRF PLA signal. Pulse‐chase experiment demonstrated a disappearance of SRPK1‐SIRF signal in H460R cells treated with 10 μm thymidine for 2 h before doing PLA. Scale bar = 5 μm. Unpaired *t*‐test. ****P < 0.0001. (B) Detection of SRPK1/TOPBP1 interaction by Proximity Ligation Assay in H460R cells treated or not (medium) for 24 h with 10 μm SPHINX31 in the presence or absence of 2 mm hydroxyurea. Scale bar = 5 μm.

**Fig. S7.** SRPK1 N‐terminal region (E43‐Q56) binds directly to TOPBP1 BRCT4/5 and BRCT7/8 domains. (A, B) Replicates of the BioLayer Interferometry (BLI) experiments showing that SRPK1‐N, either non phosphorylated or phosphorylated at serine 51, interacts with a micromolar affinity with the BRCT4/5 and BRCT7/8 domains of TOPBP1 (see also Fig. 5, panel E). Phosphorylation of serine 51 increases binding to TOPBP1 BRCT4/5, but does not increase binding to TOPBP1 BRCT7/8.

**Fig. S8.** SRPK1 does not phosphorylate AAD of TOPBP1 *in vitro*. (A) GST‐TOPBP1(978–1192) and GST‐TOPBP1(978–1522) recombinant proteins which contain RS stretches (1095–1127) inside the AAD domain of TOPBP1, or GST‐LBRNt (62–92) used as a positive control, were incubated with GST‐SRPK1 protein in an ‘*in vitro’* kinase assay. Left panel: coomassie staining of the blotted GST‐SRPK1, GST‐TOPBP1(978–1522) and GST‐TOPBP1(978–1192) proteins. Middle and right panels: autoradiograms. (B) NMR analysis of the phosphorylation of the ^15^N labeled TOPBP1 fragment AAD (residues 1055 to 1177) by SRPK1. NMR spectra were recorded on ^15^N labeled TOPBP1 (1055–1177) (50 μm, in 50 mm Hepes pH 7.0 50 mm NaCl 1 mm DTT) either alone (black) or in the presence of SRPK1 (0.14 μm), MgCl_2_ (20 mm) and ATP (5 mm). The two spectra are identical, showing that no phosphorylation is detected in our conditions.

**Fig. S9.** SPHINX31 regulates the splicing of genes involved in DNA replication and ATR signaling in NSCLC cells. (A) Number of Differentially Expressed (DEG) genes (up or down) regulated by SPHINX31 in H460S and R cells. (B) Venn diagrams of DEGs and Differentially Spliced (DSGs) genes regulated by SPHINX31 in H460S and H460R cells. Genes that are both differentially expressed and spliced are indicated for each cell line. (C) Venn diagram of DSG genes between H460S and H460R cells. Common genes between parental and resistant cells are indicated. (D) Metascape analysis of all SPHINX31‐regulated DSGs in H460S cells. (E, F) H460R cells were transfected for 72 h with either *control* (Ctrl) siRNA or two distinct siRNAs targeting *SRPK1* (Srpk1_1 and Srpk1_2). Upper panels: representative RT‐PCR agarose gels for *DBF4B* (E) or *WIZ* (F) splicing. GAPDH was used as an internal control. Numbers represent the size of amplicons in base pairs (bp). Lower panels: densitometric quantification of *DBF4B* exon 7 (E) or *WIZ* exon 8 (F) inclusion (%). Mean ± SD *n* = 3–4. (G) Left panel: representative RT‐PCR agarose gel of WIZ splice variants including or not exon 8 in A549S or R cells treated with 10 μm SPHINX31 for indicated times. GAPDH was used as an internal control. Numbers represent the size of amplicons in base pairs (bp). Right panel: densitometric quantification of WIZ splice variant retaining exon 8. Mean ± SD *n* = 3. (E, F) Unpaired *t*‐test, *P < 0.05, **P < 0.01, ***P < 0.001. (G) ANOVA two‐tailed test. ns: not significant.

**Fig. S10.** *SRPK1* and *TOPBP1*, *ATR*, *PRKDC*, *RFC5*, *DBF4B* mRNA levels and ATR/SRPK1 protein levels correlated in LUAD patients. (A) Correlations between *SRPK1* and *ATR*, *TOPBP1*, *PRKDC*, *RFC5* or *DBF4B* mRNA levels were retrieved from TCGA public database. Pearson coefficients and *P* values are indicated. (B) Correlations between *ATR* and *TOPBP1*, *PRKDC*, *RFC5* or *DBF4B* mRNA levels were retrieved from TCGA public database. Pearson coefficients and *p* values are indicated. (C) Correlation between ATR and SRPK1 protein levels was retrieved from CCLE public database. Pearson coefficients and *P* value are indicated.

**Table S1.** List of antibodies used in this study.

**Table S2.** Differentially Expressed Genes in SPHINX31‐treated H460 parental and resistant cells compared to untreated condition. Genes with *P* value ≤ 0.05 and ABS(log_2_ fold change) ≥ 0.4 were considered as significant.

**Table S3.** Differentially Spliced Genes in SPHINX31‐treated H460 parental cells compared to untreated condition. Each type of alternative splicing events is presented. Events with *P* value ≤ 0.05 and absolute deltaPSI ≥ 10% were considered as significant.

**Table S4.** Differentially Spliced Genes in SPHINX31‐treated H460 resistant cells compared to untreated condition. Each type of alternative splicing events is presented. Events with *P* value ≤ 0.05 and absolute deltaPSI ≥ 10% were considered as significant.

## Data Availability

The datasets used and analyzed during the current study are included in this published article and its Supporting Information files or available from the corresponding author on reasonable request.
